# Characteristics of protein residue-residue contacts and their application in contact prediction

**DOI:** 10.1007/s00894-014-2497-9

**Published:** 2014-11-06

**Authors:** Pawel P. Wozniak, Malgorzata Kotulska

**Affiliations:** Institute of Biomedical Engineering and Instrumentation, Wroclaw University of Technology, Wybrzeże Wyspiańskiego 27, 50-370 Wroclaw, Poland

**Keywords:** CATH, Contact propensity, Contact sites, Direct coupling analysis, Protein classification, SCOP

## Abstract

Contact sites between amino acids characterize important structural features of a protein. We investigated characteristics of contact sites in a representative set of proteins and their relations between protein class or topology. For this purpose, we used a non-redundant set of 5872 protein domains, identically categorized by CATH and SCOP databases. The proteins represented alpha, beta, and alpha+beta classes. Contact maps of protein structures were obtained for a selected set of physical distances in the main backbone and separations in protein sequences. For each set a dependency between contact degree and distance parameters was quantified. We indicated residues forming contact sites most frequently and unique amino acid pairs which created contact sites most often within each structural class. Contact characteristics of specific topologies were compared to the characteristics of their protein classes showing protein groups with a distinguished contact characteristic. We showed that our results could be used to improve the performance of recent top contact predictor — direct coupling analysis. Our work provides values of contact site propensities that can be involved in bioinformatic databases.

**Electronic supplementary material:**

The online version of this article (doi:10.1007/s00894-014-2497-9) contains supplementary material, which is available to authorized users.

## Introduction

Protein structure prediction is one of the most important topics in current bioinformatics. Information about protein tertiary structure is crucial in understanding the molecular basis of disease and can support the procedure of drug design. Another reason for protein structure prediction is low ratio of proteins with resolved structures, comparing to the total number of known protein sequences. Statistics from protein databases, UniProt [1] and Protein Data Bank (PDB) [2] (as of August 2014), show that this ratio is c.a. 0.13 %. Such a low ratio is due to the difficulties in obtaining crystallographic structures. One of the methods supporting protein structure prediction is application of contact sites prediction in the procedure of full structure prediction. Two amino acids are regarded as a contact site when two of their atoms (usually C_α_ or C_β_ from the main backbone) are not further from each other than a selected distance value. The popular measure of contact predictor performance is a prediction accuracy calculated for residue pairs which are the most probable to form the contact. Currently, the best predicting methods do not achieve accuracy higher than 60 % when considering the 200 best predicted contact sites in a protein [3–5]. This is usually insufficient for a full protein structure reconstruction. However, these numbers are still increasing along with the development of knowledge of contact sites and amino acid interactions.

In order to increase the prediction accuracy of contact prediction methods, contact characteristics in proteins must be fully examined. There have been published several studies which investigated contact characteristics within different protein groups [6–10]. Some of them also performed the analysis according to the protein classification. This is particularly important since most of the contact sites prediction methods often use protein classification in the assessment stage of the predictor design. Unfortunately, except the fact that all previous studies were based on different, not very numerous datasets, there was also no single universal way of protein classification. Many databases classify proteins differently, according to their structural or functional similarity, and organize them in different, hierarchized groups. The inconsistency of protein classification by two different databases can lead to the difficulties in comparison of methods which use them. It is not surprising then that SCOP [11] and CATH [12] databases, which became the gold-standard databases in structural biology, were extensively evaluated in terms of their differences and similarities [13, 14]. To effectively examine the contact characteristics in proteins from different structural groups, a numerous, non-redundant protein set is necessary. Moreover, the reliable classification of proteins in this set is of great importance. Only contact characteristics obtained that way will be universal and credible enough to be supportive in contact sites prediction. Furthermore, the analysis of contact characteristics in different protein structural classes can indicate subsets of proteins with unique contact distributions. These groups can be particularly interesting with respect to the assessment of contact sites prediction methods.

The first objective of our study was to examine the contact characteristics in a large, non-redundant set of protein domains which were identically classified by CATH and SCOP databases [14]. We investigated if there is any relation between characteristics of contact sites and two protein structure levels: class and topology. Class includes proteins composed mainly of one secondary structure — alpha helix or beta structure, or both in similar quantity. Topology includes proteins in which secondary structures are placed in space and inter-connected similarly to each other. Our second goal was to examine if the contact characteristics obtained this way can support the contact prediction process.

## Methods

### Contact characteristics analysis

#### Data sources

Contact sites occurrence was examined in 5872 protein domains identically categorized by CATH and SCOP databases according to Csaba et al. [14]. Proteins were classified with CATH nomenclature into three classes: alpha, beta, and alpha+beta with 1090, 1589, and 3193 proteins, respectively. We used CD-HIT [15] to ensure the sequence identity between domains was not higher than 50 % within each class. We selected 15 and 14 topologies from alpha and beta classes, respectively, which included at least 20 domains in our dataset, each. Table 1 presents a list of these topologies with their numbers later used in our analysis. Information about atom coordinates of proteins was obtained from the PDB database.

**Table 1:** List of topologies of which at least 20 proteins were available in the analyzed dataset. Topologies classification is derived from the CATH database

Number	Class	Topology
1	Alpha	Arc repressor mutant, subunit A
2	Alpha	DNA polymerase; domain 1
3	Alpha	Recoverin; domain 1
4	Alpha	Helix hairpins
5	Alpha	Globin-like
6	Alpha	Cytochrome Bc1 complex; chain D, domain 2
7	Alpha	Helicase, RuvA protein; domain 3\t
8	Alpha	Glutathione S-transferase Yfyf (Class Pi); chain A, domain 2
9	Alpha	Four helix bundle (hemerythrin (Met), subunit A)
10	Alpha	Growth hormone; Chain: A
11	Alpha	Ferritin
12	Alpha	Single alpha-helices involved in coiled-coils or other helix-helix interfaces
13	Alpha	Methane monooxygenase hydroxylase; chain G, domain 1
14	Alpha	Serine threonine protein phosphatase 5, tetratricopeptide repeat
15	Alpha	Glycosyltransferase
16	Beta	Laminin
17	Beta	Complement module; domain 1
18	Beta	Neuraminidase
19	Beta	Methylamine Dehydrogenase; Chain H
20	Beta	Pectate lyase C-like
21	Beta	PH-domain like
22	Beta	SH3 type barrels
23	Beta	Pdz3 domain
24	Beta	Lipocalin
25	Beta	Elongation factor Tu (Ef-tu); domain 3
26	Beta	OB fold (dihydrolipoamide acetyltransferase, E2P)
27	Beta	Jelly rolls
28	Beta	Immunoglobulin-like
29	Beta	Trefoil (acidic fibroblast growth factor, subunit A)

#### Contact sites parameters

To analyze the occurrence pattern of contact sites in proteins, we defined a contact site such that the space distance between C_β_ atoms of two different amino acids was not greater than a specified distance value (*cutoff*) and the contacting amino acids were separated in a protein sequence by no less than a specified number of amino acids (*separation*). In our study two adjacent residues in a sequence have *separation* equal 1. We found contact sites using *cutoff* values between 6 and 12 Å and *separation* values of 1–15 amino acids. Such *cutoff* values were chosen according to the results of Duarte et al. [16] and ensure high efficiency of protein structure reconstruction from a contact map.

#### Average contact degree

We defined *contact degree* as a number of contact sites for a specified residue. Average *contact degree* was calculated of all amino acids in analyzed proteins for specified *cutoff* and *separation* values. Dependencies between average *contact degree* and contact sites parameters (*cutoff* and *separation*) were examined. The relations were analyzed for alpha, beta, and alpha+beta classes, separately.

#### Amino acids frequency of forming contact sites

We examined the propensity of each amino acid type for contact site formation. Therefore, *W*_*c*_ parameter was defined. It shows how often, on average in all proteins, one amino acid forms a contact site related to the number of its total occurrences in proteins from a specified class. The parameter *W*_*c*_ is defined as follows:

1 $$ W{c}_{a,k}=\frac{1}{N_{a,k}}{\displaystyle \sum_{n=1}^{{}_{N_{a,k}}}\frac{I{c}_{a,n}}{I{w}_{a,n}}} $$

where:

*N*_*a,k*_: total number of proteins which contain amino acid of type *a* in their sequences and belong to class *k*
*Ic*_*a,n*_: number of amino acids of type *a* which form at least one contact site in the *n*th protein from the dataset
*Iw*_*a,n*_: number of all amino acids of type *a* in the *n*th protein from the dataset.

We examined the distribution of *W*_*c*_ values for different amino acids, separately for the results on alpha, beta, and alpha+beta classes, and applying different *cutoff* and *separation* values.

Similar to *W*_*c*_, we introduced another parameter *W*_*t*_, which shows how often one amino acid forms a contact site related to its total occurrence in proteins from specified topology. The parameter *W*_*t*_ is defined as follows:

2 $$ W{t}_{a,t}=\frac{1}{N_{a,t}}{\displaystyle \sum_{n=1}^{{}_{N_{a,t}}}\frac{I{c}_{a,n}}{I{w}_{a,n}}} $$

where:

*N*_*a,t*_: total number of proteins which contain amino acid of type *a* in their sequences and belong to topology *t*
*Ic*_*a,n*_: number of amino acids of type *a* which form at least one contact site in the *n*th protein from the dataset
*Iw*_*a,n*_: number of all amino acids of type *a* in the *n*th protein from the dataset.

#### Frequency of a contact site for a pair of amino acids

To specify amino acid pairs that create a contact site between each other the most often, the parameter *f*_*p*_ was defined. It shows how often a pair of amino acids creates a contact site, globally, summing the results from all proteins within the specific class. The *f*_*p*_ parameter is defined as follows:

3 $$ f{p}_{a_1,{a}_2,k}=\frac{J{c}_{a_1,{a}_2,k}}{J{w}_k} $$

where:

*Jc*_*a1,a2,k*_: number of contact sites formed between amino acids of types *a1* and *a2* in proteins from class *k*
*Jw*_*k*_: number of all contact sites in proteins from class *k*.

Additionally, we introduced the normalized value of parameter *f*_*p*_. It combines the information obtained from *f*_*p*_ value with the occurrence frequencies of amino acids in pair within all proteins from specific class. It is defined as follows:

4 $$ fp{n}_{a_1,{a}_2,k}=\frac{f{p}_{a_1,{a}_2,k}}{f_{a_1,k}\cdot {f}_{a_2,k}} $$

where:

*f*_*a,k*_: the occurrence frequency of amino acid of type *a* in proteins from class *k*.

The *f*_*p*_ and *f*_*pn*_ parameters were calculated for alpha, beta, and alpha+beta classes, separately. We applied *cutoff* equal 8 Å and *separation* equal ten amino acids.

#### Divergence of contact sites in different topologies

Finally, we examined the contact site characteristics within different topologies. Therefore, the parameter *S*_*t*_ was defined. *S*_*t*_ shows how similar the distribution of *W*_*t*_ values in one topology related to the distribution of *W*_*c*_ values in the class to which this topology belongs. In other words, the smaller value of *S*_*t*_, the better a topology represents its class. The *S*_*t*_ parameter is defined as follows:

5 $$ {S}_t=\sqrt{\frac{1}{N_{a,t}-1}{\displaystyle \sum_{a=1}^{N_{a,t}}{\left(\;W{t}_{a,t}-W{c}_{a,k(t)}\right)}^{\kern0.5em 2}}} $$

where:

*N*_*a,t*_: number of different amino acid types *a* which occur in topology *t* (without X amino acid — maximum 20)
*k(t)*: class *k* which contains the topology *t*
*Wc*_*a,k(t)*_: the propensity of amino acid of type *a* for contact site formation within all proteins from class *k(t)* (see Eq. 1)
*Wt*_*a,t*_: the propensity of amino acid of type *a* for contact site formation within all proteins from topology *t* (see Eq. 2).

The *S*_*t*_ parameters were examined for 29 topologies (see Table 1). We applied *cutoff* equal 10 Å and 12 Å, and *separation* equal seven amino acids.

#### Implementation

All calculation procedures were implemented in Java 1.6. Information about amino acid sequences of each CATH domain was obtained with BioJava 3.0 [17]. Visualization of the results was performed with MATLAB ver. R2011a (MathWorks).

### Application of contact characteristics in residue-residue contact prediction methods

#### Data sources

The contact sites prediction procedure presented in our study was tested on two datasets. The first one was the dataset used by us previously in Data sources to obtain contact characteristics. The second dataset was based on the protein set used by Morcos et al. [3] who used it to examine the contact sites prediction accuracy of their direct coupling analysis (DCA) algorithm. The original Morcos's set of 856 PDB structures was split into Pfam [18] database domains. Here again we used CD-HIT to ensure the sequence identity between domains was not higher than 50 %. Finally, our second dataset consisted of 562 different Pfam domains. Information about atom coordinates and secondary structures of domains were obtained from the PDB and DSSP [19, 20] databases, respectively. Multiple sequence alignment used in DCA algorithm was gained from the Pfam database.

#### Contact site definition

Contact site definition used in the assessment of contact sites prediction procedure presented in our study was based on the distance between C_β_ atoms of two different amino acids. The *cutoff* and *separation* values were 8 Å and ten amino acids, respectively.

#### Application of *f*_*p*_ parameter in residue-residue contact prediction

Our contact sites prediction procedure required the application of a main contact sites prediction method. We chose the DCA algorithm presented by Morcos et al. [3]. The DCA calculates direct information (DI) values for each of the residue pairs in the analyzed sequence. The higher value of DI, the higher probability of a pair to create a contact site. With the application of *f*_*p*_ values calculated in our study (see Eq. 3), we aimed at improving the final contact sites prediction accuracy of the DCA. The idea was to change the composition of 200 amino acid pairs with the highest DI values in each protein, so that pairs best match the *f*_*p*_ statistics calculated previously in our study. After obtaining the DI values for analyzed domain we applied the following steps:

- Each domain was assigned to one of three structural classes, alpha, beta, or alpha+beta, according to the method described by Eisenhaber et al. [21]. Therefore, basing on the data from DSSP database, residues with the secondary structural types H, G, and I were classified as helix and residues with type E were marked as sheet. Also, all helices shorter than five amino acids and strands shorter than three amino acids were reassigned to coil. Finally, according to Nakashima et al. [22], a domain that consisted of more than 15 % helices and less than 10 % sheets was classified as alpha, a domain consisted of less than 15 % helices and more than 10 % sheets was classified as beta, and a domain consisted of less than 15 % helices and less than 10 % sheets was classified as alpha+beta. A domain which did not match any of these groups was left and not examined in further steps of the procedure.
- Residue pairs were sorted according to their DI value in descending order and divided into two groups. The first group consisted of 200 pairs with the highest DI values (*top-set*) and the rest of pairs were assigned into the second group (*rest-set*).
- Starting from a pair with the lowest DI value in the *top-set* and passing along pairs with higher DI values, the *f*_*p*_ of each pair was calculated as in Eq. 6. Only amino acid pairs which included at least one residue of type X were not examined. Significant assumption was that the final improved set of residue pairs must at the end consist of 200 pairs just like the original set. Therefore, the constant value of 200 occurs in the following formulas.

6 $$ f{p}_{a_1,{a}_2,d}=\frac{J{c}_{a_1,{a}_2,d}}{200} $$

where:

*Jc*_*a1,a2,d*_: number of residue pairs including both amino acids of types *a1* and *a2* in *top-set* of domain *d*.

The *f*_*p*_ value was calculated twice for each pair. Firstly, with the occurrence of the analyzed pair in the *top-set*. Secondly, without taking the occurrence of the analyzed pair in the *top-set* into account. The constant number in Eq. 6 always equalled 200. Then, the following statement was applied:

7 $$ \begin{array}{l} if\kern0.5em \left(\;\left|f{p^{inc}}_{a_1,{a}_2,d}-f{p}_{a_1,{a}_2,k}\right|>\left|f{p^{exc}}_{a_1,{a}_2,d}-f{p}_{a_1,{a}_2,k}\right|\;\right)\kern1em  then\\ {}\kern2em  remove\kern0.5em  pair\kern0.5em  from\kern0.5em  top- set\\ {} else\\ {}\kern2em  leave\kern0.5em  pair\kern0.5em  in\kern0.5em  top- set\end{array} $$

where for the currently analyzed pair containing amino acids of types *a1* and *a2*:

*fp*^*inc*^_*a1,a2,d*_: *f*_*p*_ value for the residue pairs containing amino acids of types *a1* and *a2* in the *top-set* of domain *d* (see Eq. 6) including (*inc*) the occurrence of the analyzed pair in the *top-set*
*fp*^*exc*^_*a1,a2,d*_: *f*_*p*_ value for the residue pairs containing amino acids of types *a1* and *a2* in the *top-set* of domain *d* (see Eq. 6) excluding (*exc*) the occurrence of the analyzed pair in the *top-set*
*fp*_*a1,a2,k*_: *f*_*p*_ value for the residue pairs containing amino acids of types *a1* and *a2* (see Eq. 3) for class *k* to which the analyzed domain belongs according to the step a).

The removed pairs created the third group — *rmv-set*. Residue pairs in *rmv-set* were sorted by their assignment into this set.

d) Starting from a pair with the highest DI value in the *rest-set* and passing along pairs with lower DI values, the *f*_*p*_ of each pair was calculated as in Eq. 6. This value was again calculated twice for each pair (with and without the occurrence of the analyzed pair in the *top-set*) and not for amino acid pairs which consisted of at least one residue type X. The constant number in Eq. 6 still equals 200. Then, the following statement was applied:

8 $$ \begin{array}{l} if\kern0.5em \left(\kern0.5em \left|f{p^{inc}}_{a_1,{a}_2,d}-f{p}_{a_1,{a}_2,k}\right|>\left|f{p^{exc}}_{a_1,{a}_2,d}-f{p}_{a_1,{a}_2,k}\right|\;\right)\kern1em  the n\\ {}\kern2em  leave\kern0.5em  pair\kern0.5em  in\kern0.5em  rest- set\\ {} else\\ {}\kern2em  add\kern0.5em  pair\kern0.5em to\kern0.5em  the\kern0.5em  end\kern0.5em  of\kern0.5em  top- set\end{array} $$

where for the analyzed pair containing amino acids of types *a1* and *a2*:

*fp*^*inc*^_*a1,a2,d*_: *f*_*p*_ value for the residue pairs containing amino acids of types *a1* and *a2* in the *top-set* of domain *d* (see Eq. 6) including (*inc*) the occurrence of the analyzed pair in the *top-set*
*fp*^*exc*^_*a1,a2,d*_: *f*_*p*_ value for the residue pairs containing amino acids of types *a1* and *a2* in the *top-set* of domain *d* (see Eq. 6) excluding (*exc*) the occurrence of the analyzed pair in the *top-set*
*fp*_*a1,a2,k*_: *f*_*p*_ value for the residue pairs containing amino acids of types *a1* and *a2* (see Eq. 3) for class *k* to which the analyzed domain belongs according to the step a).

The procedure described above was performed until the number of pairs in the *top-set* was equal to 200 or until all pairs in the *rest-set* were examined.

e) This step was executed only if number of pairs in the *top-set* was not equal to 200 after the previous steps. Then, until this number was equal to 200, the latest added pairs in *rmv-set* were inserted at the end of the *top-set*.

We examined the results of our procedure for all domains in the dataset at once and separating them into classes. The algorithm assessment was performed as in Morcos et al. [3]. Therefore, we calculated the average true positive (TP) rate of contact prediction in analyzed domains as a function of the number of top-ranked contacts from 1 to 200.

#### Application of *f*_*pn*_ parameter in residue-residue contact prediction

The procedure presented in Application of fp parameter in residue-residue contact prediction was repeated with the application of *f*_*pn*_ values calculated in our study (see Eq. 4). Therefore, Eq. 6 was replaced as follows:

9 $$ fp{n}_{a_1,{a}_2,d}=\frac{\frac{J{c}_{a_1,{a}_2,d}}{200}}{f_{a_1,d}\cdot {f}_{a_2,d}} $$

where:

*Jc*_*a1,a2,d*_: number of residue pairs consisting both amino acids of types *a1* and *a2* in *top-set* of domain *d*
*f*_*a,d*_: the occurrence frequency of amino acid of type *a* in domain *d*.

Constant value equal to 200 occurred in Eq. 9 for the same reason as it was in Eq. 6. The final improved set of residue pairs must at the end consist of 200 pairs just like the original set.

Then, Eq. 7 was changed into the following equation:

10 $$ \begin{array}{l} if\kern0.5em \left(\;\left|fp{n^{inc}}_{a_1,{a}_2,d}-fp{n}_{a_1,{a}_2,k}\right|>\left|fp{n^{exc}}_{a_1,{a}_2,d}-fp{n}_{a_1,{a}_2,k}\right|\;\right)\kern1em  then\\ {}\kern2em  remove\kern0.5em  pair\kern0.5em  from\kern0.5em  top- set\\ {} else\\ {}\kern2em  leave\kern0.5em  pair\kern0.5em  in\kern0.5em  top- set\end{array} $$

where for the analyzed pair containing amino acids of types *a1* and *a2*:

*fpn*^*inc*^_*a1,a2,d*_: normalized value of *f*_*p*_ for the residue pairs containing amino acids of types *a1* and *a2* in the *top-set* of domain *d* (see Eq. 9) including (*inc*) the occurrence of the analyzed pair in the *top-set*
*fpn*^*exc*^_*a1,a2,d*_: normalized value of *f*_*p*_ for the residue pairs containing amino acids of types *a1* and *a2* in the *top-set* of domain *d* (see Eq. 9) excluding (*exc*) the occurrence of the analyzed pair in the *top-set*
*fpn*_*a1,a2,k*_: normalized value of *f*_*p*_ for the residue pairs containing amino acids of types *a1* and *a2* (see Eq. 4) for class *k* to which the analyzed domain belongs according to the step a) in Application of fp parameter in residue-residue contact prediction.

Finally, Eq. 8 was replaced with:

11 $$ \begin{array}{l} if\kern0.5em \left(\;\left|fp{n^{inc}}_{a_1,{a}_2,d}-fp{n}_{a_1,{a}_2,k}\right|>\left|fp{n^{exc}}_{a_1,{a}_2,d}-fp{n}_{a_1,{a}_2,k}\right|\;\right)\kern1em  the n\\ {}\kern2em  leave\kern0.5em  pair\kern0.5em  in\kern0.5em  rest- set\\ {} else\\ {}\kern2em  add\kern0.5em  pair\kern0.5em to\kern0.5em  the\kern0.5em  end\kern0.5em  of\kern0.5em  top- set\end{array} $$

where for the analyzed pair containing amino acids of types *a1* and *a2*:

*fpn*^*inc*^_*a1,a2,d*_: normalized value of *f*_*p*_ for the residue pairs containing amino acids of types *a1* and *a2* in the *top-set* of domain *d* (see Eq. 9) including (*inc*) the occurrence of the analyzed pair in the *top-set*
*fpn*^*exc*^_*a1,a2,d*_: normalized value of *f*_*p*_ for the residue pairs containing amino acids of types *a1* and *a2* in the *top-set* of domain *d* (see Eq. 9) excluding (*exc*) the occurrence of the analyzed pair in the *top-set*
*fpn*_*a1,a2,k*_: normalized value of *f*_*p*_ for the residue pairs containing amino acids of types *a1* and *a2* (see Eq. 4) for class *k* to which the analyzed domain belongs according to the step a) in Application of fp parameter in residue-residue contact prediction.

#### Implementation

Contact sites prediction procedure presented in our study was implemented in Java 1.6. The DCA algorithm was adopted in Java 1.6 as described by Morcos et al. [3]. The implementation of DCA in Java was tested and compared with its original implementation in MATLAB (http://dca.upmc.fr/DCA/DCA.html). Visualization of the results was performed with MATLAB ver. R2011a (MathWorks).

## Results

### Contact characteristics analysis

#### Dataset representativity

**Fig. 1:**
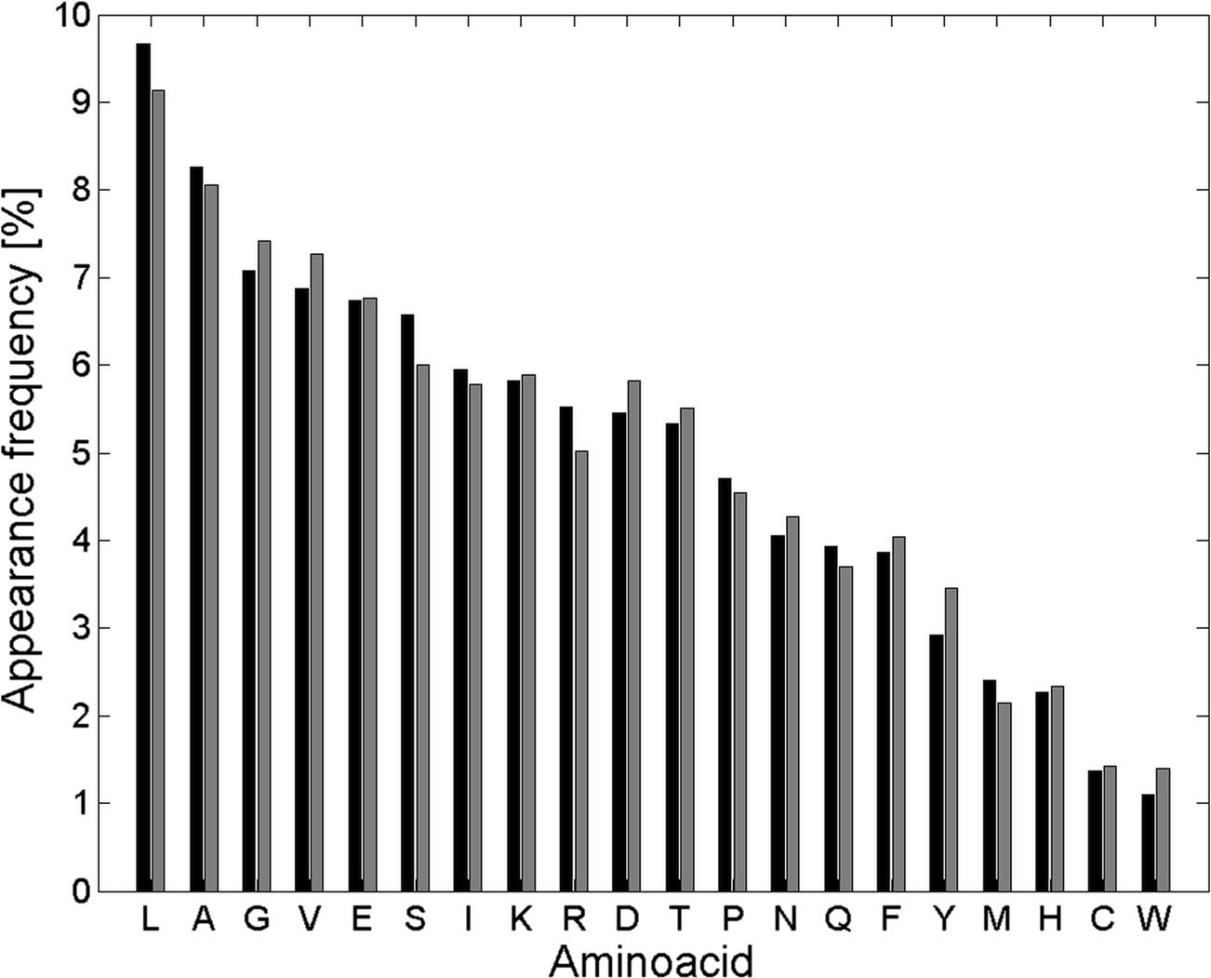
Frequency of amino acids in proteins from UniProt database (*black*) and our database (*gray*)

First, we analyzed if our basic dataset (see Data sources) is representative of the whole protein world. Figure 1 compares the frequency between amino acids in proteins from UniProt database (as of August 2014) and analyzed in this work.

Figure 1 shows that similarity between frequency distributions for both datasets is high. First six amino acids with the highest values of appearance frequency are the same. Furthermore, two-sample Kolmogorov-Smirnov tests did not reject the hypothesis that presented frequencies from both analyzed datasets were from the same continuous distribution (5 % significance). Kendall rank correlation coefficient for distributions from Fig. 1 equalled 0.94, which means that they are highly correlated. This proves that our analyzed dataset is representative and consistent with proteins collected in UniProt database.

#### Average contact degree

**Fig. 2:**
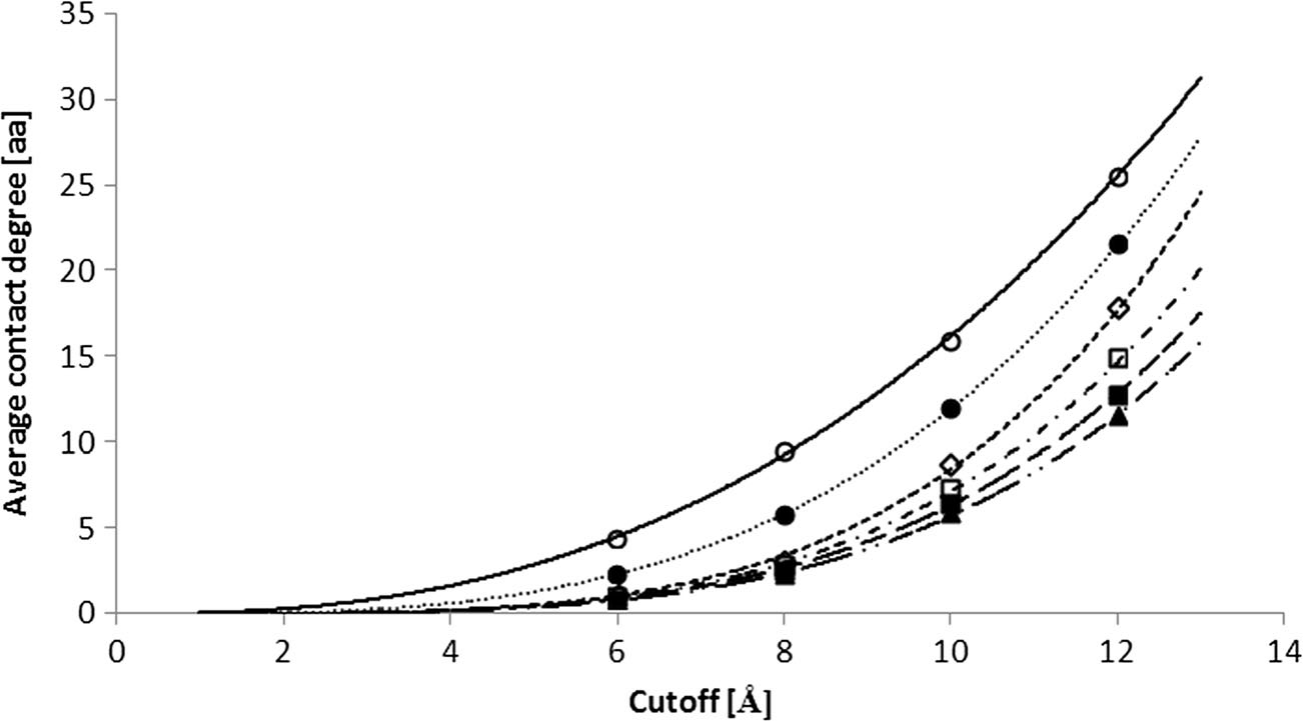
Average *contact degree* of proteins from class alpha as a function of *cutoff* used in contact sites definition. Plots presented for different *separations*: one (*white circle*), three (*black circle*), five (*white diamond*), seven (*white square*), ten (*black square*), and fifteen (*black up-pointing triangle*) amino acids. Plots are fitted with power functions specified in Table 2

Figure 2 shows the dependency between average *contact degree* (see Average contact degree) and *cutoff* distance used in contact sites definition for proteins form alpha class. The analysis was carried out for different values of *separation*.

All presented dependencies are power regardless of the *separation* parameter for alpha (Fig. 2), beta (not shown), and alpha+beta (not shown) classes. Table 2 shows values of *a* and *b* parameters of the fitting function *y = a∙x* ^*b*^, which best matched both classes. In the table we also present R-squared values, which account for the accuracy of every interpolated function. These values are always higher than 0.99, which represents high interpolation matching.

**Table 2:** Values of *a* and *b* parameters (and the R-squared values of interpolations) from the fitting function *y = a∙x* ^*b*^ for alpha, beta, and alpha+beta classes

	Alpha			Beta			Alpha+Beta		
Separation	a	b	R^2^	a	b	R^2^	a	b	R^2^
1	0.0514	2.50	0.999	0.0454	2.56	0.999	0.0447	2.59	1.000
3	0.0073	3.22	1.000	0.0059	3.31	1.000	0.0065	3.31	1.000
5	0.0007	4.06	0.997	0.0046	3.36	1.000	0.0023	3.66	1.000
7	0.0008	3.95	0.999	0.0042	3.37	1.000	0.0023	3.63	1.000
10	0.0009	3.87	0.999	0.0036	3.40	1.000	0.0022	3.61	1.000
15	0.0007	3.89	0.999	0.0027	3.47	1.000	0.0019	3.62	1.000

**Fig. 3:**
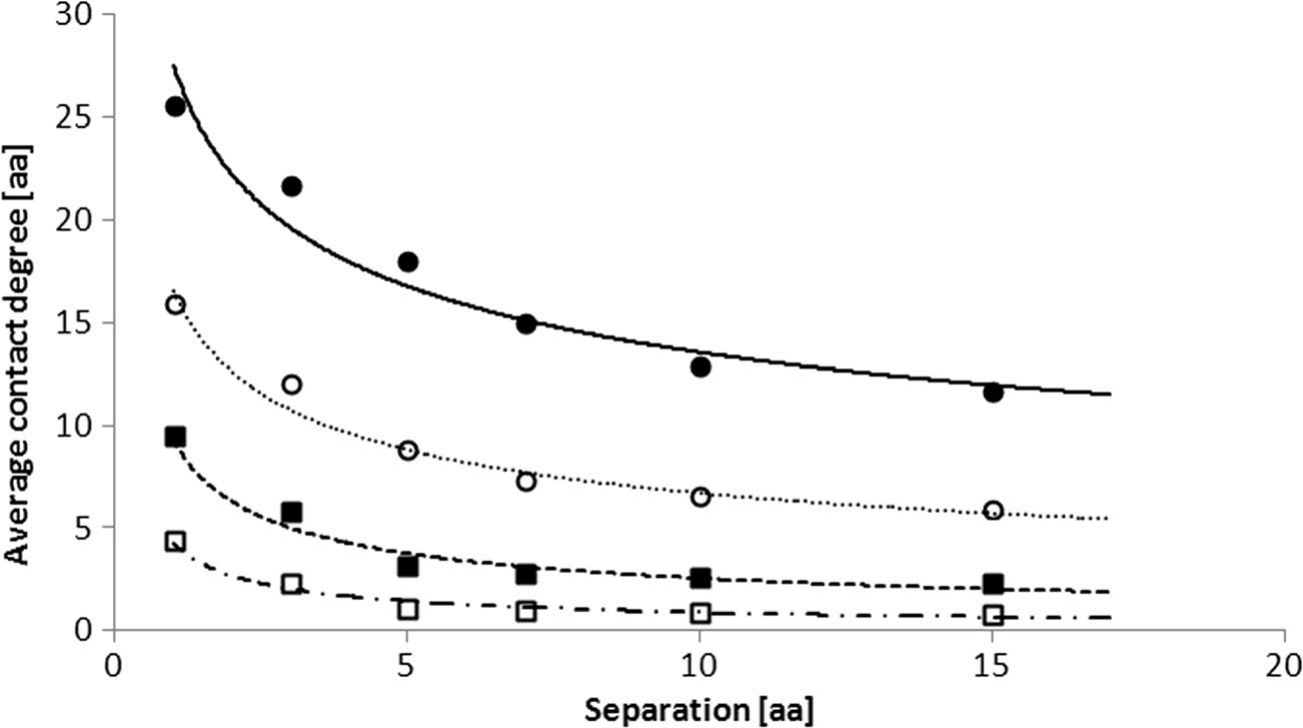
Average *contact degree* of proteins from class alpha as a function of *separation* used in contact sites definition. Plots presented for different *cutoffs*: 6 Å (*white square*), 8 Å (*black square*), 10 Å (*white circle*), and 12 Å (*black circle*). Plots are fitted with power functions

Figure 3 shows the dependency between average *contact degree* and *separation* used in contact sites definition for proteins from alpha class. The analysis was studied for different *cutoff* values. The shapes of the plots were similar with the results for proteins from beta and alpha+beta classes (not shown). Again each plot can be interpolated with power function — *a* and *b* parameters of the fitting function *y = a∙x* ^*b*^ are shown in Table 3. R-squared values of these plots interpolations are always higher than 0.94, therefore the power interpolation is fully acceptable. However, we note that standard deviation of the points in Figs. 2 and 3 is 30–140 % for class alpha, 35-100 % for class beta, and 35-110 % for class alpha+beta of their average value.

**Table 3:** Values of *a* and *b* parameters (and the R-squared values of interpolations) from the fitting function *y = a∙x* ^*b*^ for alpha, beta, and alpha+beta classes

	Alpha			Beta			Alpha+Beta		
Cutoff	a	b	R^2^	a	b	R^2^	a	b	R^2^
6	4.26	−0.68	0.943	3.95	−0.42	0.955	4.25	−0.49	0.958
8	9.30	−0.57	0.947	9.01	−0.32	0.979	9.54	−0.39	0.970
10	16.6	−0.39	0.975	15.9	−0.25	0.999	17.4	−0.29	0.992
12	27.6	−0.31	0.950	26.6	−0.20	0.990	29.3	−0.23	0.974

Tables 2 and 3 show that average *contact degree* is always higher for proteins from class beta than those from class alpha, regardless of the *cutoff* and *separation* values. It is due to a higher density of β-sheets than that of α-helices. Furthermore, Figs. 2 and 3 show that while the dependency between average *contact degree* increases with the *cutoff* value, average *contact degree* decreases with the *separation* value. Moreover, the slope of the dependency between average *contact degree* and *cutoff* value is steeper than that of the average *contact degree* and *separation* value. It is particularly notable for *separation* higher than three amino acids. There can be several reasons explaining the difference in slopes. Firstly, the change in the *cutoff* value should have a higher impact on contact site occurrence. Since the average atom size equals 1 Å, the average dimension of amino acid backbone is about 3 Å. Therefore, to make a change of *separation* parameter more important for the occurrence of contact site, *separation* parameter should be less than three amino acids for *cutoff* value of 6 Å and less than five amino acids for *cutoff* value of 12 Å.

#### Amino acids frequency of forming contact sites

**Fig. 4:**
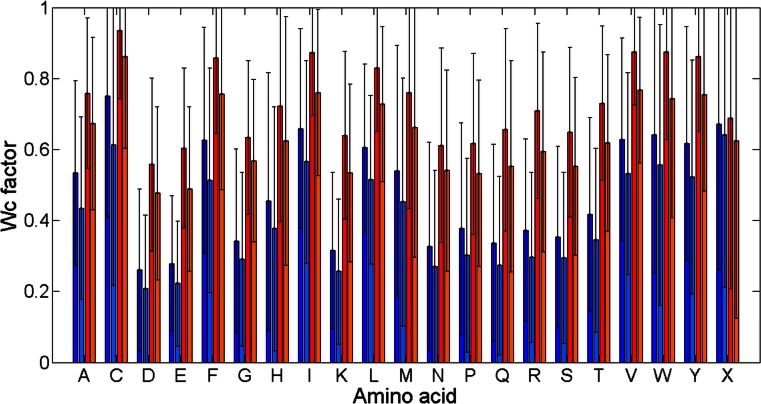
Values of *W*_*c*_ for different amino acid types at *cutoff* 6 Å. Class alpha and *separation* 7 (*dark blue*), class alpha and *separation* 15 (*bright blue*), class beta and *separation* 7 (*red*), class beta and *separation* 15 (*orange*). Standard deviations for every *W*_*c*_ value were added to the figure

**Fig. 5:**
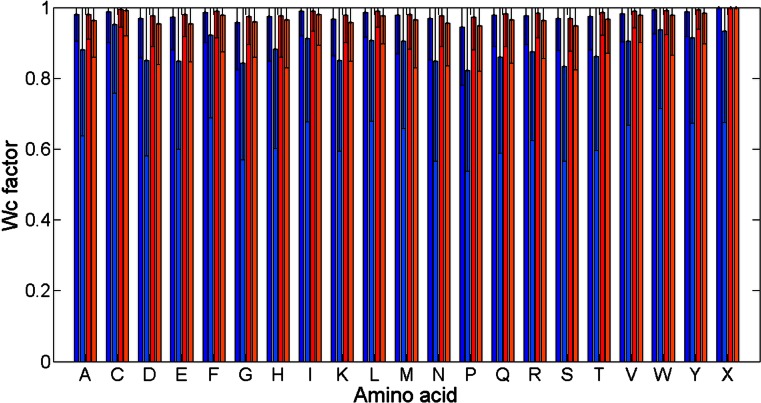
Values of *W*_*c*_ for different amino acid types at *cutoff* 12 Å. Class alpha and *separation* 7 (*dark blue*), class alpha and *separation* 15 (*bright blue*), class beta and *separation* 7 (*red*), class beta and *separation* 15 (*orange*). Standard deviations for every *W*_*c*_ value were added to the figure

Figures 4 and 5 present bar plots of *W*_*c*_ values (see Amino acids frequency of forming contact sites, Eq. 1) for different amino acid types and contact sites parameters. Comparison between results for class alpha and results for class beta shows that their characteristics are similar. The only difference is a higher level reached by bars for class beta. It stems from the fact that the occurrence of contact sites in class beta is higher than in class alpha. Furthermore, the change of *cutoff* and *separation* parameters affects *W*_*c*_ values for all amino acids in the same way. With the increase of the *cutoff* values, the *W*_*c*_ values grow for all amino acids. The difference between bar heights for two adjoining amino acids on the x-axis remains the same. However, similarly to what was observed in the previous paragraph, the change of the *separation* has lower impact on contact sites occurrence than a change of the *cutoff.*

Finally, Figs. 4 and 5 give a possibility of indicating amino acids with particularly high levels of *W*_*c*_ value. They are similar for both alpha and beta classes: Cys, Ile, Leu, Met, Phe, Trp, Tyr, and Val. The reason why these amino acids create contact sites the most often lays in their physicochemical nature. All of them are hydrophobic and neutral. The lack of charge makes them more prone to make a contact site because they are not repelled by amino acids with a positive or negative charge. Therefore, they can form contact sites with more amino acids. Furthermore, hydrophobicity is greatly related to the burial of the residue in the protein structure. In general, in water environment, hydrophobic amino acids are pushed away from water molecules to inside of the protein. Hence, they have more close amino acid neighbors, which makes them more favorable in forming contact site. This direct relation between residue accessibility and its propensity to create contact site was already described by Faure et al. [10] and is compatible with results shown in Figs. 4 and 5. Finally, from all amino acids cysteine creates contact sites the most often. Besides the fact that this residue is on average the most buried amino acid [10], it has the ability of creating disulfide bonds which take part in the protein tertiary structure creation. Cysteines in such bonds, which are in the same time far from each other in the sequence, are thought to be a contact site. Since most of the cysteines create this bond, their *W*_*c*_ value is particularly high.

Similarly, we can indicate amino acids whose *W*_*c*_ values are the lowest. These are Asp, Glu, and Lys, which are all hydrophilic and charged.

The values of *W*_*c*_ parameters for all amino acid types, both alpha and beta classes and chosen contact site parameters (*cutoff*: 6 Å, 8 Å, and 12 Å, *separation*: 5 and 15) are presented in Table A.1 and Table A.2 in Appendix A. Additionally, Table A.3 in Appendix A contains *W*_*c*_ parameters for alpha+beta class and the same contact site parameters as for alpha and beta classes.

#### Frequency of a contact site for a pair of amino acids

We analyzed the top 20 residue pairs which create contact sites the most and the least often in the analyzed proteins from classes alpha and beta, separately. The *f*_*p*_ values (see Frequency of a contact site for a pair of amino acids, Eq. 3) of all amino acid pairs in these classes are presented in the upper halves of Table B.1 and Table B.2 (Appendix B). According to Figs. 4 and 5, the amino acid types which create contact sites the most often (high *W*_*c*_ values) mostly appear within top creating contact sites pairs (Table B.1). Domination of leucine within top pairs from Table B.1 and Table B.2 is due to the fact that leucine occurs in the analyzed protein dataset the most often (Fig. 1). Therefore, the probability of creating the contact site between leucine and other amino acids is the highest. On the other hand, cysteine does not appear within top pairs from Table B.1, despite the high *W*_*c*_ value, because it occurs in the analyzed protein dataset the least often.

Top 20 pairs from alpha and beta classes in upper halves of Table B.1 and Table B.2 share 13 residue pairs. However, analyzing alpha and beta classes individually, unique top pairs for each class can be pointed out. These are for example Ala-Ala in class alpha and Val-Val in class beta. Probably it stems from the propensities of these pairs to certain secondary structures. For example, alanine is one of the most popular amino acids within proteins of class alpha, because of its high propensity to α-helices. Similarly, valine prefers to lie within β-sheets and it is difficult for this amino acid to adopt the α-helical conformation [23].

In the top 20 residue pairs creating contact sites the least often, different 11 amino acid pairs can be pointed out, occurring in both alpha and beta classes. The most common residues within these pairs are cysteine and tryptophan. It is because both cysteine and tryptophan are amino acids which appear the least often within all amino acids in proteins from analyzed dataset (Fig. 1).

Due to the observation of the strong dependency between the *f*_*p*_ values and the occurrence frequency of different amino acid types, the parameter *f*_*p*_ was normalized. The *f*_*pn*_ values (see Frequency of a contact site for a pair of amino acids, Eq. 4) of all amino acid pairs are presented in lower halves of Table B.1 and Table B.2 (Appendix B). The results show that a domination of leucine within top creating contact sites pairs is not only related to its high occurrence in the analyzed dataset. After normalization leucine still presents high propensity for a contact site creation. However, Table B.1 and Table B.2 show that now amino acids with the highest contact sites propensities are also Cys, Phe, Trp, and Met which had the lowest values of parameter *f*_*p*_ before normalization. It perfectly matches the results presented in Figs. 4 and 5 and is connected with the physicochemical nature of these amino acids. The highest value obtained for Cys-Cys pair in both alpha and beta classes suggests that the ability of cysteine to create disulfide bonds is greatly connected with its high propensity to creating contact sites. On the other hand, there are much fewer differences in top creating contact sites pairs between classes alpha and beta than it was for parameter *f*_*p*_. However, still some distinctions can be observed. Results obtained for class alpha present more pairs containing cysteine and methionine, while results for class beta show more pairs containing tryptophan within top creating contact sites pairs.

The *f*_*p*_ and *f*_*pn*_ values of all amino acid pairs in class alpha+beta were not compared in detail with results for classes alpha and beta in our study. However, they are presented in Table B.3 (Appendix B) and will be used later in our analysis.

**Fig. 6:**
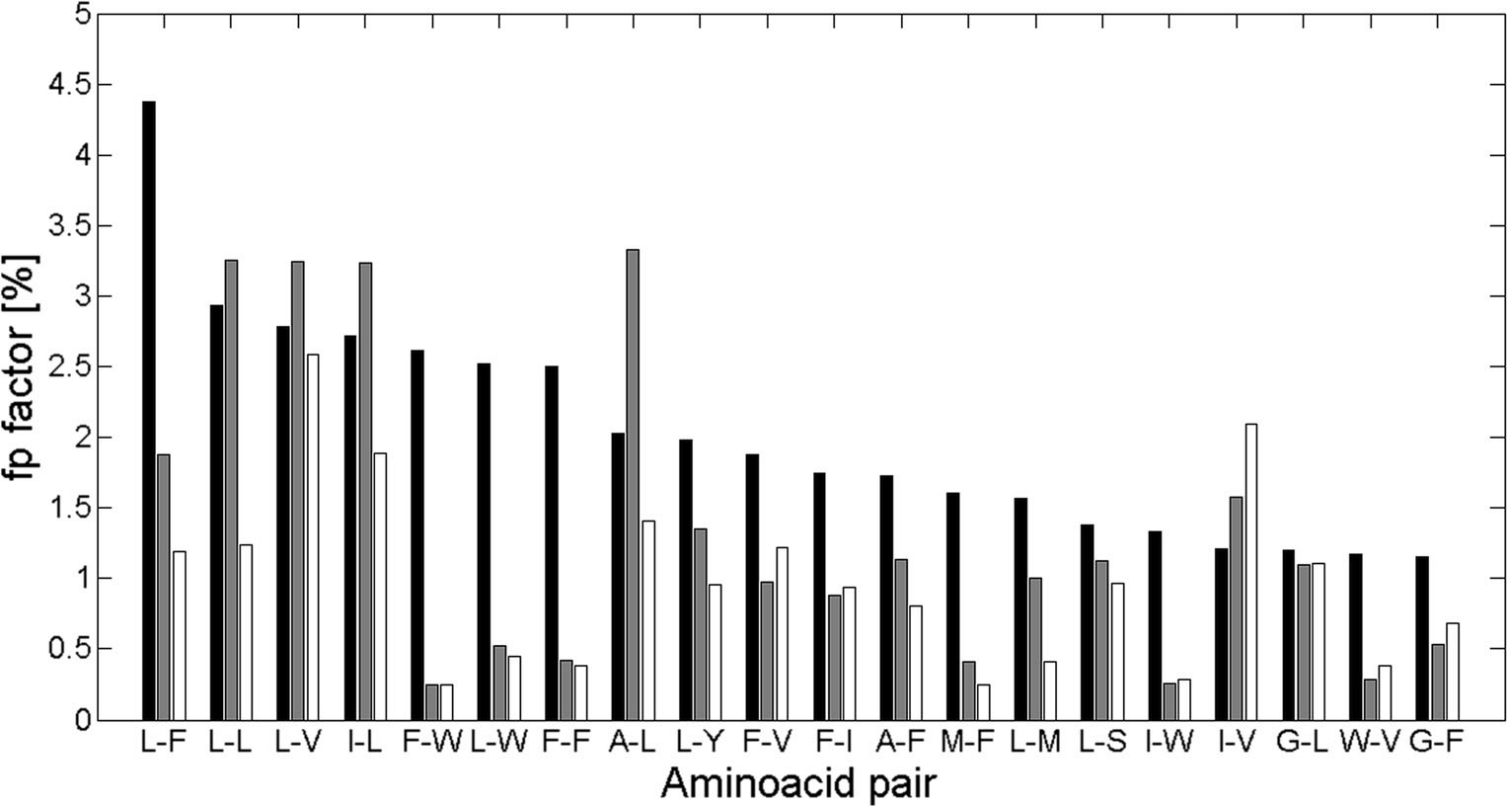
Frequency for the top 20 interacting pairs of *membrane* proteins from Adamian and Liang [9] (*black bins*) with our *f*_*p*_ factors of these pairs from proteins from class alpha (*gray bins*) and class beta (*white bins*). Here *cutoff* is 8 Å and *separation* is 10

**Fig. 7:**
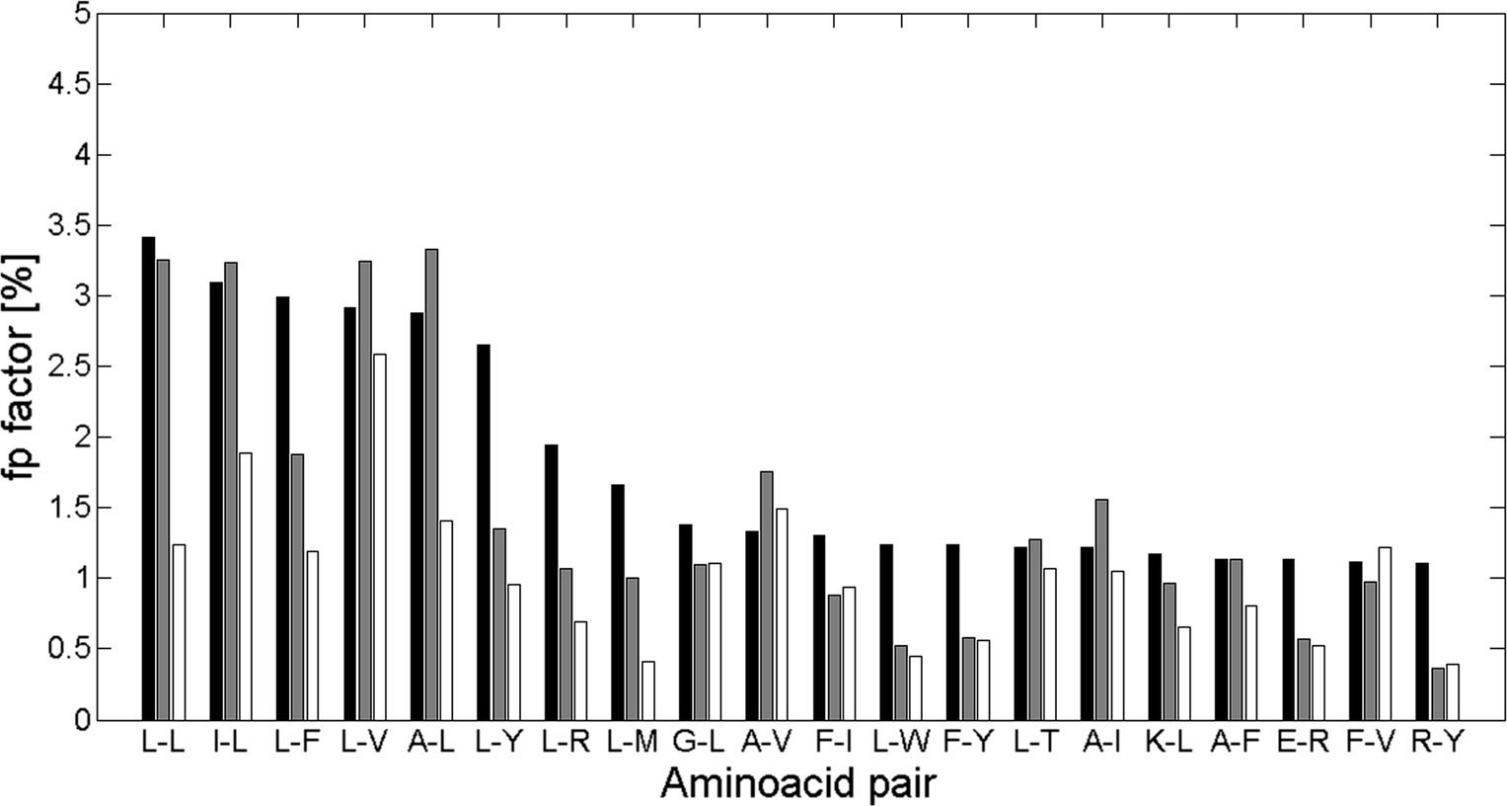
Frequency for the top 20 interacting pairs of *soluble* proteins from Adamian and Liang [9] (*black bins*) with our *f*_*p*_ factors of these pairs from proteins from class alpha (*gray bins*) and class beta (*white bins*). Here *cutoff* is 8 Å and *separation* is 10

Analysis of residue pairs propensities to create contact sites appeared in other studies before [6–10]. Despite significant differences in compositions and sizes of datasets, the most distinctive difference between methodologies applied in those studies is a contact site definition. Depending on the definition used, the results were more similar to *f*_*p*_ or *f*_*pn*_ parameter presented in our study. Therefore, we examined the similarity of the results obtained while applying different contact definition, dataset composition, and dataset size comparing to the results of our analysis. Thus, we compared our results with the results of Adamian and Liang [9]. Adamian and Liang presented frequencies of top 20 interacting residue pairs analyzed in 14 membrane proteins and 31 soluble alpha-helical proteins. These values are presented in Figs. 6 and 7 with the *f*_*p*_ values of amino acid pairs for proteins from our dataset, for classes alpha and beta separately. Numbers representing the bars on the plots are presented in Table C.1 (Appendix C). It is significant that the number of proteins used by Adamian and Liang in their analysis is much lower than in our study and that their definition of interaction somehow differs from our definition of contact site. They define the interaction using the geometric structures of the Voronoi diagram, the Delaunay triangulation and the alpha complex. Contacting atoms are the atoms whose Voronoi cells intersect. Nevertheless, despite the differences in contact site definition and the size of the analyzed datasets, Figs. 6 and 7 show important similarities.

As Table C.1 shows, *f*_*p*_ values for amino acid pairs for proteins from class alpha are more similar to the top 20 interacting pairs frequencies from membrane and soluble proteins, than those from class beta. It stems from the fact that both sets of membrane and soluble proteins contain mainly alpha helices, which are the main structures of alpha class proteins. Furthermore, *f*_*p*_ values for pairs from class alpha match top 20 interacting pairs from soluble proteins better than those from membrane proteins. This occurs probably because soluble proteins contain some small amount of non-helical substructures, like most of proteins from class alpha, but still having alpha-helical structures in majority.

Finally, comparing Table B.1 and Table B.2 with Table C.1 it can be observed that most of the pairs within the top 20 from Table B.1 and Table B.2 belong also to the top 20 interacting pairs from Adamian and Liang results. Twelve and ten pairs in membrane proteins are also within the top 20 pairs from alpha and beta class proteins, respectively. Also, 15 and 11 top soluble pairs belong to top alpha class and top beta class proteins, appropriately. The amino acid which occurs in most of the shared pairs is leucine. All these pairs are denoted in bold in Table C.1.

#### Divergence of contact sites in different topologies

**Fig. 8:**
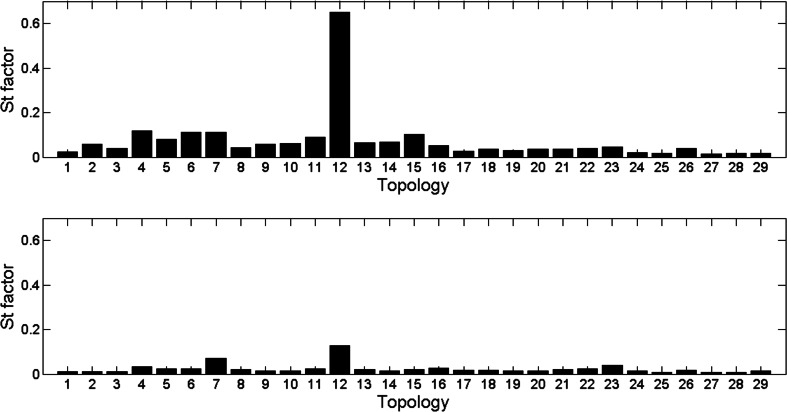
Values of *S*_*t*_ in analyzed topologies for *separation* 7 and *cutoff* 10 Å (*upper plot*) and 12 Å (*lower plot*)

**Fig. 9:**
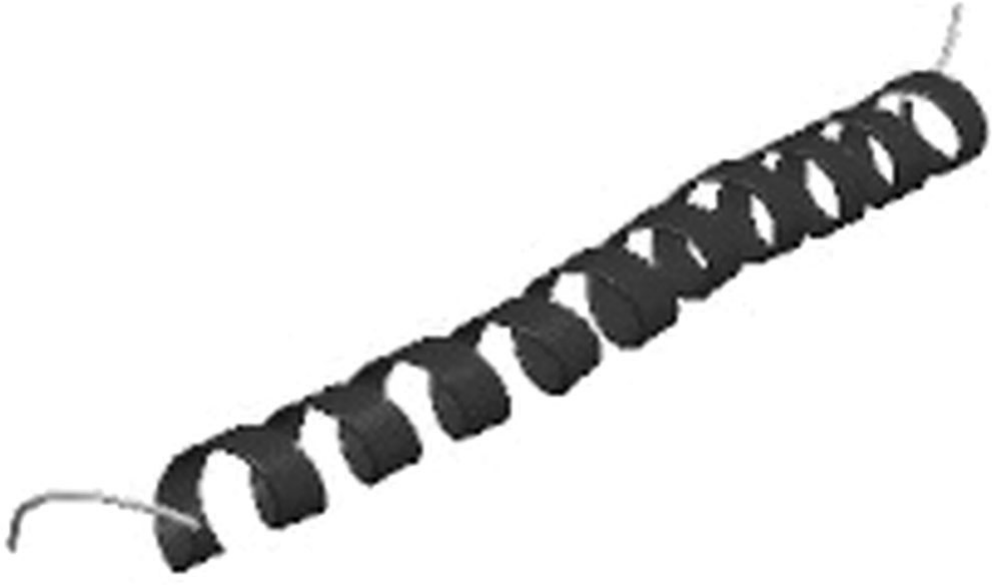
Example domain 1mz9A00 of 12th topology “Single alpha-helices involved in coiled-coils or other helix-helix interfaces” [12]

In the end, we examined the frequency of contact sites for different amino acids in different topologies. Figure 8 presents *S*_*t*_ values (see Divergence of contact sites in different topologies, Eq. 5) for all 29 topologies (see Data sources, Table 1) for the *cutoff* of 10 Å and 12 Å, separately, and *separation* of seven amino acids. Figure 8 shows that most of the topologies properly represent their classes (*S*_*t*_ value lower than 0.1). However, the 12th topology from class alpha (“Single alpha-helices involved in coiled-coils or other helix-helix interfaces”) has *S*_*t*_ much higher than the other topologies for *cutoff* of 10 Å. It means that its contact site characteristic differs from its class. This topology includes proteins of single alpha helices (an example domain is shown in Fig. 9). Therefore, it is difficult to obtain any contact site in this kind of protein, especially with the *separation* value of seven. For *separation* of seven amino acids the differences between *S*_*t*_ values of compared topologies were the best to observe, which can be explained with the fact that this *separation* is equal to the double of α-helice period. However, some of the proteins in the 12th topology have contact sites because of the bends in their α-helices. Moreover, the comparison of *S*_*t*_ values for *cutoff* of 10 Å and *cutoff* of 12 Å shows that, for *cutoff* of 12 Å, difference between 12th topology and its class lowers (its *S*_*t*_ value decreases). It probably stems from the fact that 12 Å is a distance far enough to count amino acids farther than 3 periods in α-helice as contact sites.

### Application of contact characteristics in residue-residue contact prediction methods

We examined if contact characteristics parameters described in our study can support recent contact sites prediction methods. We chose the *f*_*p*_ value which represents the frequency of a residue pair to form a contact site and its normalized version — the *f*_*pn*_. These parameters were the most distinctive in analyzed protein structural classes.

#### Application of *f*_*p*_ parameter in residue-residue contact prediction

**Fig. 10:**
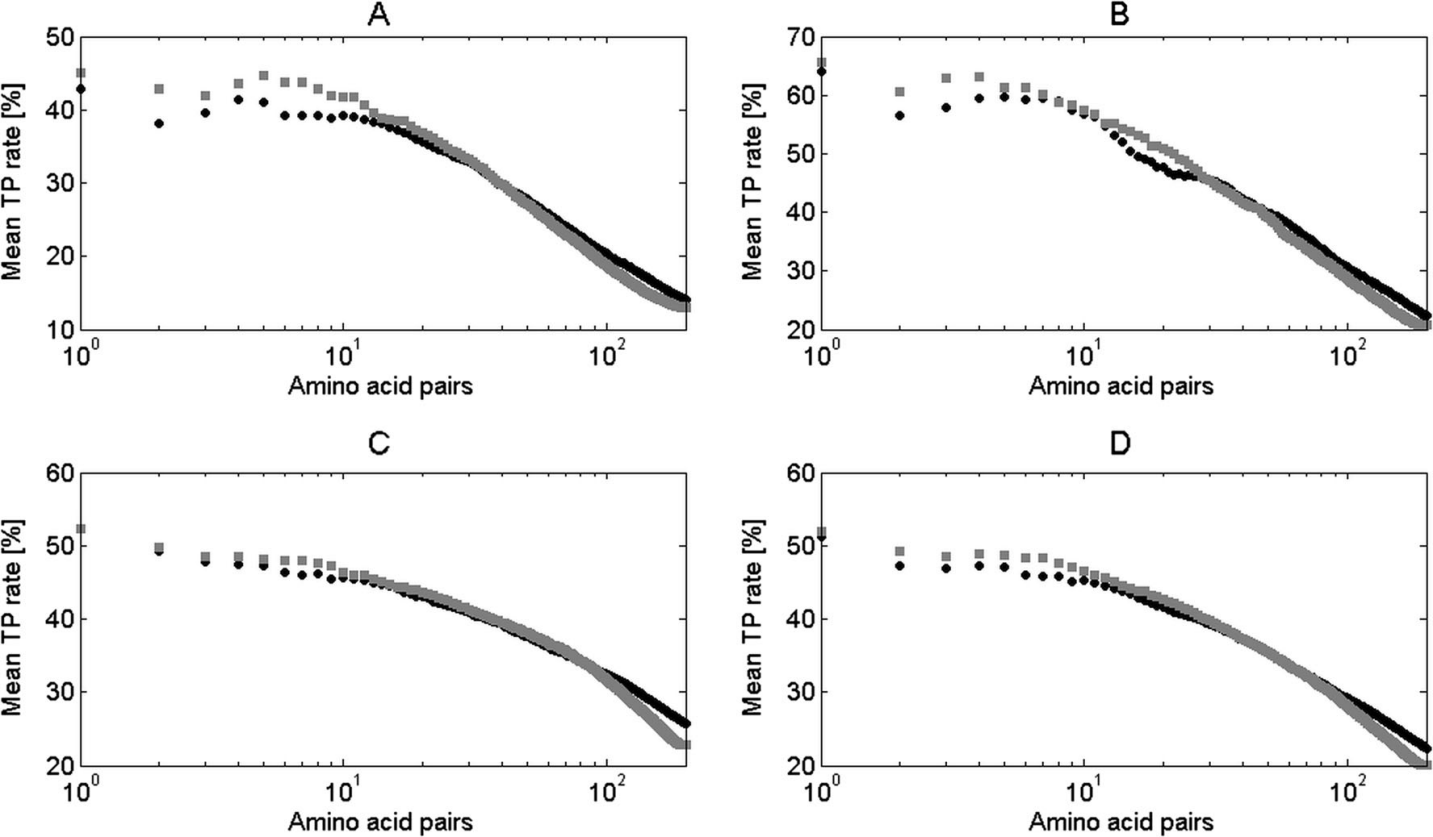
Mean TP rate for different number of top-ranked contacts in proteins from Morcos et al. [3] dataset. Results for original DCA algorithm (*black circle*) and with application of *f*_*p*_ (*gray square*) are presented for: **a** domains from class alpha, **b** domains from class beta, **c** domains from class alpha+beta, **d** all domains

Figure 10 shows the results of application of the *f*_*p*_ value in improving the contact site prediction accuracy of the DCA algorithm (see Application of fp parameter in residue-residue contact prediction). The analysis was done for the dataset based on that used by Morcos et al. [3] (see Data sources).

Figure 10 shows that the application of *f*_*p*_ value in contact sites prediction algorithm influenced the results for alpha and beta classes more clearly than the results for alpha+beta class. There is a high improvement for the ten top-ranked contacts in these classes. In fact, the numbers show that better contact prediction accuracy was achieved for up to 40, 50, and 100 top-ranked pairs in alpha, beta, and alpha+beta classes, respectively. The result obtained for all classes (Fig. 10d) greatly resembles the one for alpha+beta class (Fig. 10c). This is probably related to the highest number of domains examined in this class, which dominate in the set of all domains. Also, there is a slight decrease of mean TP rate when more than 100 top-ranked contacts are analyzed. This means that while *f*_*p*_ value can usually properly eliminate non-contact pairs within pairs with the highest DCA results, it is not that successful while adding new pairs into the top 200 pairs. It is also connected with a progressive drop of contact prediction accuracy of DCA algorithm with the increase of top-ranked pairs. Choosing a residue pair to add it into new top 200 pairs from previously not assigned pairs, our algorithm starts with pairs with the highest DCA result. Contact prediction accuracy of this result for the 200th pair and lower is only on the level of about 25 %.

**Fig. 11:**
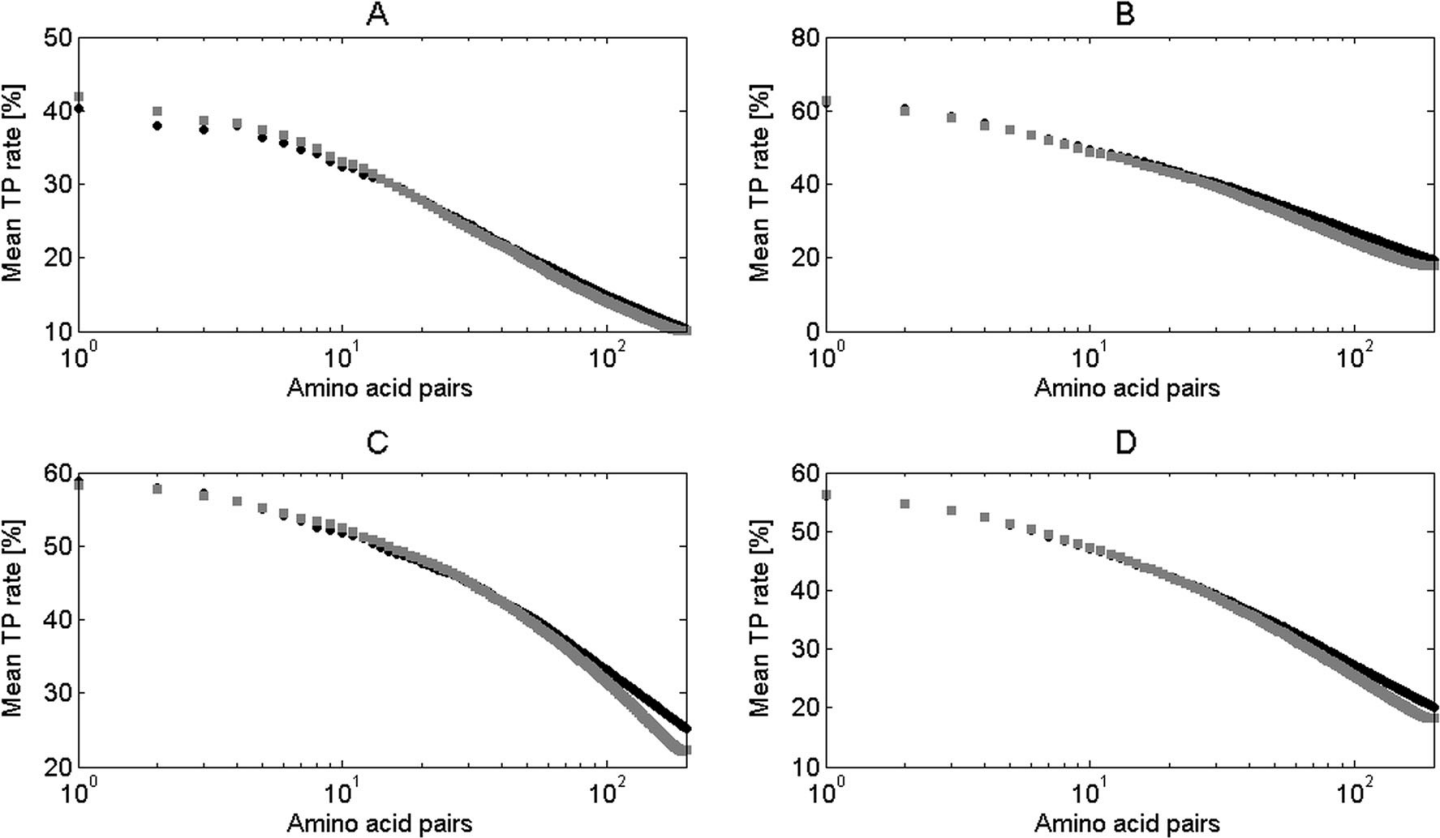
Mean TP rate for different number of top-ranked contacts in proteins from dataset used to calculate the *f*_*p*_ value in our study. Results for original DCA algorithm (*black circle*) and with application of *f*_*p*_ (*gray square*) are presented for: **a** domains from class alpha, **b** domains from class beta, **c** domains from class alpha+beta, **d** all domains

We compared the results from Fig. 10 obtained for Morcos et al. [3] dataset with the results gained for the dataset used to calculate the *f*_*p*_ value in our study. These are presented in Fig. 11. In this case, the improvement for the top 50 contacts is negligible and the contact sites prediction accuracy stays at the similar level after the application of the *f*_*p*_ value. Even previously observed decrease of TP rate for more than 100 top-ranked pairs is much lower. However, still the best results were obtained for the domains from alpha class. Results presented in Fig. 11 suggest that our algorithm performs better for the more specific dataset. Data used by Morcos et al. [3] came from mainly bacterial domain families with large non-redundant multiple sequence alignments. Domains examined in our study do not belong to any specific protein family but can be clearly assigned to one structural group. This shows that presented algorithm is dataset source-dependant.

#### Application of *f*_*pn*_ parameter in residue-residue contact prediction

**Fig. 12:**
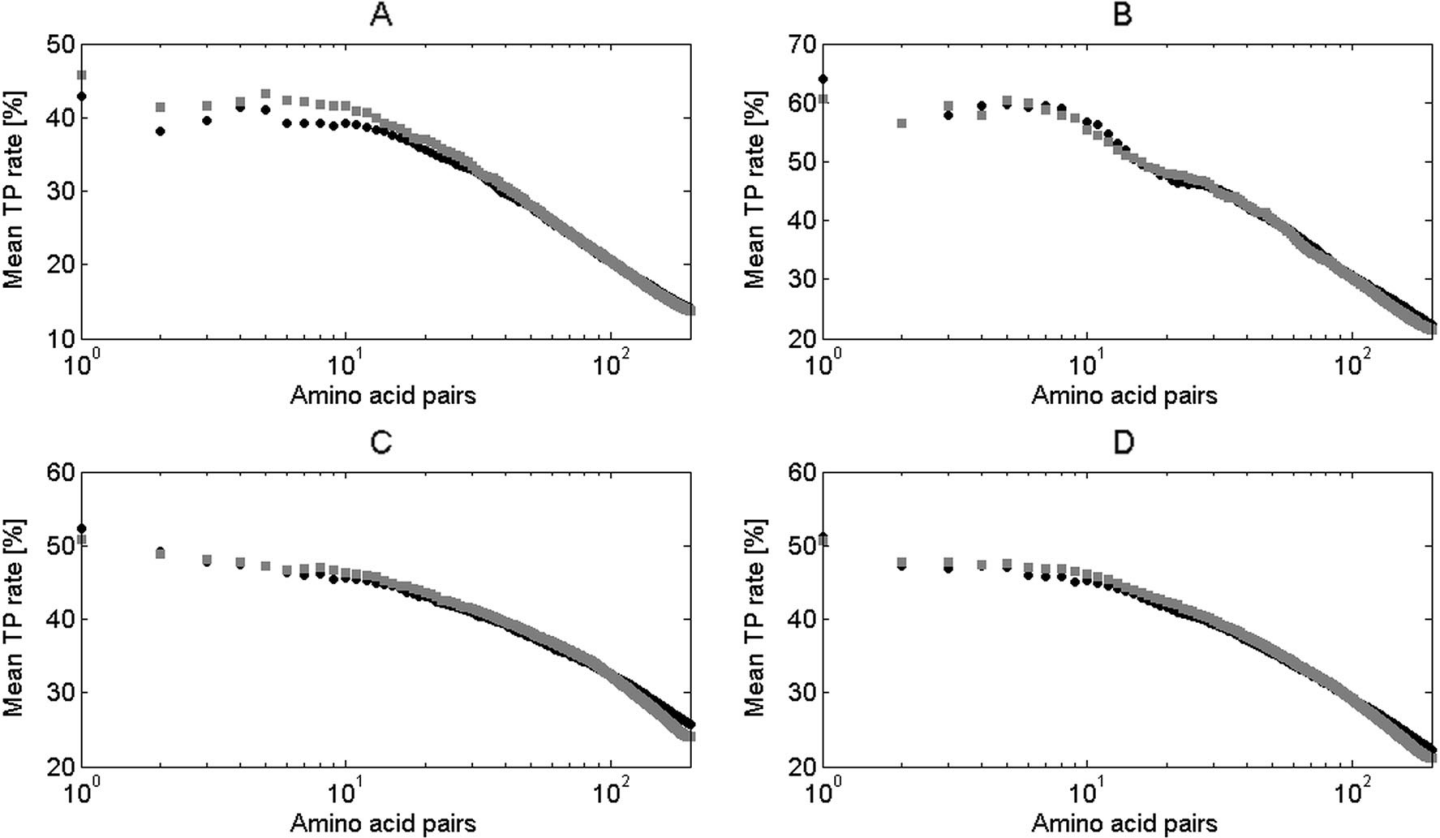
Mean TP rate for different number of top-ranked contacts in proteins from Morcos et al. [3] dataset. Results for original DCA algorithm (*black circle*) and with application of *f*_*pn*_ (*gray square*) are presented for: **a** domains from class alpha, **b** domains from class beta, **c** domains from class alpha+beta, **d** all domains

Finally, we examined how the application of *f*_*pn*_ value in contact sites prediction influences the prediction accuracy of DCA algorithm (see Application of fpn parameter in residue-residue contact prediction). The results for the dataset based on that used by Morcos et al. [3] (see Data sources) are presented in Fig. 12. There is much lower improvement in the prediction when the *f*_*pn*_ value is applied comparing to the previous results achieved for the application of the *f*_*p*_ value (Fig. 10). However, the increase in mean TP rate for domains from alpha class is still evident. This result probably stems from the fact that the *f*_*p*_ value was much more distinctive for alpha and beta classes than the *f*_*pn*_ value (see Frequency of a contact site for a pair of amino acids). The *f*_*pn*_ value eliminates information coming from the occurrence frequency of amino acid types in different protein structural classes. On the other hand, Fig. 12 shows that there is almost no decrease of accuracy when more than 100 top-ranked contacts are analyzed. This is related to the fact that the algorithm based on the *f*_*pn*_ value eliminates fewer residue pairs with the highest DCA results. As a result, it also adds fewer new pairs into the improved 200 top pairs. Therefore, the final mean TP rate plot looks much more similar to the original DCA algorithm plot, comparing with the effect of *f*_*p*_ value application.

We again examined the influence of the *f*_*pn*_ value on contact sites prediction in domains from our dataset. The results were similar to those observed in Fig. 11 for the *f*_*p*_ value, therefore they were not presented. The only difference was a smaller decrease of TP rate when more than 100 top-ranked contacts are analyzed.

## Discussion and conclusions

Our study introduced terms and parameters which can support contact sites prediction or the assessment of contact predictors. We showed that there is a dependency between the average *contact degree* and distance parameters (*cutoff* and *separation*), which could be fitted with the power function. Moreover, we proposed the parameters *W*_*c*_ and *W*_*t*_ which specified amino acid types the most prone to create a contact site within specific class or topology. Values of *f*_*p*_ and *f*_*pn*_ enabled the exhaustive insight into pairs creating contact sites the most and the least often. It gave the possibility of specifying the unique pairs for each analyzed protein class. At this point, we showed that despite a different definition of a contact site used by Adamian and Liang [9] and a significantly lower number of instances they analyzed, there was a significant resemblance between their and our results. By introducing the *S*_*t*_ factor, we also showed that within different protein classes there might be topologies with totally different characteristics and frequencies of contact sites occurrence, while comparing to the characteristics of their classes. The *S*_*t*_ factor is dependent on contact sites parameter values. Finally, we showed that with the application of introduced parameters (*f*_*p*_ and *f*_*pn*_) we could improve the contact sites prediction accuracy of one of the top contact sites predictors — the DCA algorithm.

Csaba et al. [14] reported many significant differences in protein classification between two most respected protein structure-based classification databases — SCOP and CATH. This inconsistence can lead to serious problems in protein studies, e.g., while comparing methods tested on different datasets. It also includes contact sites prediction methods which very often examine their prediction accuracy on different structural protein groups. It shows that the universal way of protein classification is needed. In case of contact sites prediction methods, a protein classification database which divides proteins according to their contact characteristics might be helpful. We believe that such a database would have a strong application in the assessment and comparison of contact sites predictors. One of the steps of creating protein classification tools is to represent a protein sequence with a numerical model [24]. Parameters presented in our study, such as *f*_*p*_ *, f*_*pn*_, or *W*_*c*_ *,* point residues with distinguishing propensities of creating contact sites in different protein groups and thus can be used in creation of such a model. Furthermore, the introduced *S*_*t*_ factor can specify protein topologies which do not match their classes in the meaning of contact sites occurrence, indicating structural groups with unique contact characteristics.

One of the typical assumptions in contact sites prediction is that the number of contact sites in one protein is directly proportional to its sequence length [25]. This rule is frequently used while evaluating the accuracy of the contact sites predictors [26–30]. A statistical model calculated after the prediction of contact sites can provide the information if the number of predicted contact sites is proper. It can also indicate if a group of predicted contact sites perturbs the expected global statistics of all predicted contact sites for one protein. For example, if the average *contact degree* or the distribution of the *W*_*c*_ factor is very far from the expected characteristic, then there might be a possibility that some of the contact sites were badly predicted. The observation of protein average *contact degree* after contact sites prediction can suggest if the global prediction result is proper. Furthermore, despite the fact that relations between frequencies of forming contact sites by different amino acids were similar in both alpha and beta classes, it is possible to indicate amino acids whose propensities to create contact sites are particularly high for one of those classes. It is mainly related to their physicochemical nature and can also be supportive in the contact sites prediction process.

In our study we showed that the application of the simple algorithm based only on *f*_*p*_ or *f*_*pn*_ parameters could improve the prediction accuracy of one of the top recent contact predictors — the DCA algorithm. It is probable that the already achieved result can be even better after combining these parameters with the other introduced in our analysis, like *contact degree* or parameter *W*_*c*_. Also, there are various factors which could affect the performance of our algorithm. Firstly, we obtained the information about the protein structural classification basing on a simple method described by Eisenhaber et al. [21] and Nakashima et al. [22]. However, there are many other protein structural class predictors which can be used and achieve even better accuracy [31–33]. Secondly, we applied the *f*_*p*_ and *f*_*pn*_ parameters to improve the performance of the DCA algorithm. The choice of the main prediction algorithm is of great importance. We used DCA because it has been one of the top contact sites prediction method recently. The obtained results were satisfactory but there might be even better effect achieved with the application of different predicting method. For example, we observed that the prediction accuracy of DCA for more than 200 best predicted contact pairs was only at the level of 25 %. This significantly affected the performance of our method since we used these pairs in creation of a new set of 200 best predicted pairs. Another important factor, which had an impact on the performance of our method, was the source of the tested dataset. We showed that much higher improvement was obtained for a dataset of Morcos et al. [3] than for our dataset used in the analysis of contact characteristics. This first group of domains was much more specific since it contained mainly bacterial proteins, while the second one was bigger and more general. The dependency between the source of tested data and the performance of contact sites prediction methods is well known. Nevertheless our method showed some improvement of a chosen contact predictor accuracy. Especially when the analyzed data is not representative enough or when the results are divided into different structural classes. Finally, the performance of our method can also be dependent on a number of amino acids of type X in a sequence, since these residues were not analyzed in our algorithm. Therefore, they were not removed from the initially predicted set of the 200 best predicted pairs.

A high demand for the numerical biological data has been common recently. One of the most popular databases, gathering the information about various physicochemical and biochemical properties, expressed in numbers, is AAindex [34]. By typing the word *contact* in its browser it outputs various information such as the interaction energies between side chains of different amino acids [35] or the measure of the exposure of a residue to solvent [36]. There are only single results containing data related to the subject of the contact sites and protein classification [8]. The results presented in our study can support the AAindex database. Values of the propensities of different amino acid types to create contact sites (*W*_*c*_ factors), attributed to different structural classes, are presented in Appendix A of our work. Also, the values of parameter *f*_*p*_, before and after the normalization, are shown in Appendix B. This data is a ready-to-use set of values that can be included into the AAindex resources.

In the previous years there were other studies on the propensities of creating contact sites by different amino acids [6–10]. We compared our results with Adamian and Liang because of distinct differences between contact definition, dataset composition, and dataset size. Their results were consistent with our work. However, there were also other studies which applied different methodology than ours. In the studies published in [6–8] contact propensities were represented by the effective contact energies between residues. The energies were obtained from the numbers of contacts observed in experimental studies. In [6–8] contact site definition was based on the distance between the centers of the side chain atoms (usually C_β_) and the *cutoff* value equal 6.5 Å. It was shown that there is a high similarity between intra- and intermolecular contact energies. Zhang and Kim [8], whose results can be found in the AAindex, also provided the data about the influence of the secondary structures on the inter-residue interactions [8]. Unfortunately, in the year 2000 significantly fewer protein structures were known than currently, thus the datasets were not very numerous (Zhang and Kim used only 407 selected protein domains). Since the validity of a statistical survey depends on the size of the dataset [37], we analyzed almost 6000 non-redundant protein domains (sequence identity not higher than 50 %) in our study. The results of contact propensities based on the contact energies were comparable with those received in our study — presented as normalized parameters *f*_*p*_ in lower halves of the Tables in Appendix B. The propensity of residues to create contact sites was also examined by Faure et al. [10]. Their definition was closer to our *f*_*pn*_ parameter than those based on contact energies. The main difference is that Faure et al. analyzed preferential contacts of amino acid types in a different manner, in which the order of amino acids mattered and there could be different values of relative contact frequency for residue pairs such as Ala-Leu and Leu-Ala. Despite these differences, our results show some qualitative similarities which are, for example, a high contact propensity of cysteine or affinities between certain residues. However, there is still a significant difference in size and the composition of datasets used in both studies. Faure et al. examined about 1200 protein chains with 10 % pairwise sequence identity while our dataset consisted of almost 6000 protein domains with 50 % sequence identity. Moreover, our domains were those identically classified by SCOP and CATH databases and Faure et al. used only SCOP classification. Summing up, we confirmed the previously reported results, despite the differences in contact site definition, size of the dataset, and methods used. Our study presents an insight into the subject of amino acids propensities to the creation of contact sites based on the most recent datasets and is compatible with the previous studies. Presented results show the possibility of their application in the process of contact predictors assessment or contact site prediction.

## Electronic supplementary material

Below is the link to the electronic supplementary material.

ESM 1 (DOCX 25 kb)

ESM 2 (DOCX 38 kb)

ESM 3 (DOCX 20 kb)
