# Supplementary material for: Characteristics of protein residue-residue contacts and their application in contact prediction
Source: J Mol Model. 2014 Nov 6;20(11):2497. doi: 10.1007/s00894-014-2497-9 (PMC4221654; doi:10.1007/s00894-014-2497-9)
Supplement: Supplementary file 1 — (DOCX 25 kb) [file 894_2014_2497_MOESM1_ESM.docx]

**Appendix A**

Table A.1 Values of *W_c_* factors of different amino acid types in proteins from class Alpha. Here *cutoffs* are: 6 Å, 8 Å and 12 Å; *separations* are 5 and 15

|  |  | **Alpha** | | | | | |
| --- | --- | --- | --- | --- | --- | --- | --- |
|  | **Separation** | **5 amino acids** | | | **15 amino acids** | | |
|  | **Cutoff** | **6 Å** | **8 Å** | **12 Å** | **6 Å** | **8 Å** | **12 Å** |
| **Amino acid type** | **A** | 0.57 | 0.72 | 0.99 | 0.43 | 0.59 | 0.88 |
|  | **C** | 0.80 | 0.91 | 1.00 | 0.61 | 0.80 | 0.95 |
|  | **D** | 0.31 | 0.58 | 0.99 | 0.21 | 0.43 | 0.85 |
|  | **E** | 0.32 | 0.58 | 0.99 | 0.22 | 0.44 | 0.85 |
|  | **F** | 0.68 | 0.86 | 0.99 | 0.51 | 0.78 | 0.92 |
|  | **G** | 0.41 | 0.67 | 0.98 | 0.29 | 0.51 | 0.84 |
|  | **H** | 0.50 | 0.73 | 0.99 | 0.38 | 0.59 | 0.88 |
|  | **I** | 0.72 | 0.87 | 0.99 | 0.57 | 0.78 | 0.91 |
|  | **K** | 0.37 | 0.63 | 0.99 | 0.26 | 0.47 | 0.85 |
|  | **L** | 0.68 | 0.86 | 1.00 | 0.51 | 0.77 | 0.91 |
|  | **M** | 0.61 | 0.82 | 0.99 | 0.45 | 0.71 | 0.90 |
|  | **N** | 0.37 | 0.61 | 0.99 | 0.27 | 0.46 | 0.85 |
|  | **P** | 0.42 | 0.64 | 0.98 | 0.30 | 0.48 | 0.82 |
|  | **Q** | 0.39 | 0.65 | 0.99 | 0.27 | 0.50 | 0.86 |
|  | **R** | 0.43 | 0.70 | 0.99 | 0.30 | 0.54 | 0.87 |
|  | **S** | 0.40 | 0.63 | 0.99 | 0.29 | 0.48 | 0.83 |
|  | **T** | 0.46 | 0.69 | 0.99 | 0.35 | 0.54 | 0.86 |
|  | **V** | 0.68 | 0.84 | 0.99 | 0.53 | 0.73 | 0.91 |
|  | **W** | 0.68 | 0.88 | 1.00 | 0.56 | 0.80 | 0.94 |
|  | **Y** | 0.67 | 0.85 | 1.00 | 0.52 | 0.75 | 0.92 |
|  | **X** | 0.67 | 0.88 | 1.00 | 0.64 | 0.83 | 0.93 |

Table A.2 Values of *W_c_* factors of different amino acid types in proteins from class Beta. Here *cutoffs* are: 6 Å, 8 Å and 12 Å; *separations* are 5 and 15

|  |  | **Beta** | | | | | |
| --- | --- | --- | --- | --- | --- | --- | --- |
|  | **Separation** | **5 amino acids** | | | **15 amino acids** | | |
|  | **Cutoff** | **6 Å** | **8 Å** | **12 Å** | **6 Å** | **8 Å** | **12 Å** |
| **Amino acid type** | **A** | 0.79 | 0.89 | 0.99 | 0.67 | 0.81 | 0.96 |
|  | **C** | 0.95 | 0.98 | 1.00 | 0.86 | 0.94 | 0.99 |
|  | **D** | 0.60 | 0.81 | 0.99 | 0.48 | 0.70 | 0.95 |
|  | **E** | 0.65 | 0.84 | 0.99 | 0.49 | 0.71 | 0.95 |
|  | **F** | 0.88 | 0.96 | 0.99 | 0.76 | 0.89 | 0.98 |
|  | **G** | 0.67 | 0.84 | 0.99 | 0.57 | 0.76 | 0.96 |
|  | **H** | 0.76 | 0.90 | 0.98 | 0.62 | 0.81 | 0.97 |
|  | **I** | 0.90 | 0.96 | 0.99 | 0.76 | 0.90 | 0.98 |
|  | **K** | 0.68 | 0.87 | 0.99 | 0.53 | 0.75 | 0.96 |
|  | **L** | 0.86 | 0.95 | 0.99 | 0.73 | 0.89 | 0.98 |
|  | **M** | 0.79 | 0.92 | 0.99 | 0.66 | 0.85 | 0.96 |
|  | **N** | 0.65 | 0.85 | 0.99 | 0.54 | 0.75 | 0.96 |
|  | **P** | 0.65 | 0.86 | 0.98 | 0.53 | 0.76 | 0.95 |
|  | **Q** | 0.70 | 0.87 | 0.99 | 0.55 | 0.75 | 0.96 |
|  | **R** | 0.75 | 0.90 | 0.99 | 0.59 | 0.79 | 0.96 |
|  | **S** | 0.68 | 0.84 | 0.98 | 0.55 | 0.74 | 0.95 |
|  | **T** | 0.77 | 0.90 | 0.99 | 0.62 | 0.79 | 0.97 |
|  | **V** | 0.90 | 0.96 | 0.99 | 0.77 | 0.89 | 0.98 |
|  | **W** | 0.89 | 0.96 | 1.00 | 0.74 | 0.90 | 0.98 |
|  | **Y** | 0.89 | 0.96 | 1.00 | 0.75 | 0.89 | 0.98 |
|  | **X** | 0.69 | 0.88 | 1.00 | 0.63 | 0.81 | 1.00 |

Table A.3 Values of *W_c_* factors of different amino acid types in proteins from class Alpha+Beta. Here *cutoffs* are: 6 Å, 8 Å and 12 Å; *separations* are 5 and 15

|  |  | **Alpha+Beta** | | | | | |
| --- | --- | --- | --- | --- | --- | --- | --- |
|  | **Separation** | **5 amino acids** | | | **15 amino acids** | | |
|  | **Cutoff** | **6 Å** | **8 Å** | **12 Å** | **6 Å** | **8 Å** | **12 Å** |
| **Amino acid type** | **A** | 0.74 | 0.85 | 0.99 | 0.64 | 0.78 | 0.96 |
|  | **C** | 0.91 | 0.97 | 1.00 | 0.80 | 0.92 | 0.99 |
|  | **D** | 0.52 | 0.75 | 0.99 | 0.42 | 0.65 | 0.95 |
|  | **E** | 0.49 | 0.72 | 0.99 | 0.38 | 0.61 | 0.95 |
|  | **F** | 0.82 | 0.95 | 1.00 | 0.70 | 0.88 | 0.98 |
|  | **G** | 0.63 | 0.83 | 0.99 | 0.54 | 0.74 | 0.96 |
|  | **H** | 0.68 | 0.86 | 0.99 | 0.58 | 0.78 | 0.96 |
|  | **I** | 0.86 | 0.96 | 1.00 | 0.76 | 0.91 | 0.98 |
|  | **K** | 0.56 | 0.79 | 0.99 | 0.44 | 0.67 | 0.95 |
|  | **L** | 0.80 | 0.94 | 1.00 | 0.67 | 0.88 | 0.98 |
|  | **M** | 0.76 | 0.91 | 0.99 | 0.63 | 0.83 | 0.96 |
|  | **N** | 0.58 | 0.79 | 0.99 | 0.48 | 0.69 | 0.95 |
|  | **P** | 0.61 | 0.81 | 0.99 | 0.52 | 0.72 | 0.95 |
|  | **Q** | 0.55 | 0.79 | 1.00 | 0.43 | 0.67 | 0.96 |
|  | **R** | 0.62 | 0.84 | 0.99 | 0.49 | 0.72 | 0.96 |
|  | **S** | 0.62 | 0.81 | 0.99 | 0.51 | 0.71 | 0.95 |
|  | **T** | 0.70 | 0.87 | 0.99 | 0.59 | 0.77 | 0.96 |
|  | **V** | 0.87 | 0.95 | 1.00 | 0.77 | 0.89 | 0.98 |
|  | **W** | 0.79 | 0.93 | 1.00 | 0.66 | 0.86 | 0.98 |
|  | **Y** | 0.80 | 0.93 | 1.00 | 0.68 | 0.86 | 0.98 |
|  | **X** | 0.81 | 0.92 | 0.96 | 0.72 | 0.86 | 0.95 |
