# Supplementary material for: Characteristics of protein residue-residue contacts and their application in contact prediction
Source: J Mol Model. 2014 Nov 6;20(11):2497. doi: 10.1007/s00894-014-2497-9 (PMC4221654; doi:10.1007/s00894-014-2497-9)
Supplement: Supplementary file 2 — (DOCX 38 kb) [file 894_2014_2497_MOESM2_ESM.docx]

**Appendix B**

Table B.1 Parameters *f_p_* (Upper Half) and *f_pn_* (Lower Half) for all amino acid pairs in proteins from class Alpha. Top 20 residue pairs with the highest values are in bold and the 20 residue pairs with the lowest values are underlined. Here the *cutoff* values is 8 Å and the *separation* value is 10

|  | **A** | **C** | **D** | **E** | **F** | **G** | **H** | **I** | **K** | **L** | **M** | **N** | **P** | **Q** | **R** | **S** | **T** | **V** | **W** | **Y** | **f_p_** |  |  |
| --- | --- | --- | --- | --- | --- | --- | --- | --- | --- | --- | --- | --- | --- | --- | --- | --- | --- | --- | --- | --- | --- | --- | --- |
|  | **1.30** | 0.40 | 0.58 | 0.77 | **1.14** | **1.14** | 0.43 | **1.56** | 0.63 | **3.33** | 0.63 | 0.55 | 0.61 | 0.56 | 0.72 | 0.93 | 0.93 | **1.75** | 0.36 | 0.91 | **A** |  |  |
| **A** | 1.69 | 0.18 | 0.10 | 0.12 | 0.26 | 0.20 | 0.11 | 0.33 | 0.13 | 0.64 | 0.12 | 0.10 | 0.13 | 0.12 | 0.15 | 0.19 | 0.17 | 0.33 | 0.06 | 0.19 | **C** |  |  |
| **C** | 3.31 | **9.36** | 0.13 | 0.25 | 0.28 | 0.39 | 0.24 | 0.31 | 0.44 | 0.61 | 0.14 | 0.28 | 0.31 | 0.22 | 0.51 | 0.40 | 0.35 | 0.39 | 0.12 | 0.26 | **D** |  |  |
| **D** | 1.16 | 1.26 | 0.38 | 0.15 | 0.40 | 0.40 | 0.23 | 0.53 | 0.53 | **1.06** | 0.22 | 0.28 | 0.32 | 0.28 | 0.57 | 0.44 | 0.39 | 0.53 | 0.14 | 0.33 | **E** |  |  |
| **E** | 1.18 | 1.21 | 0.58 | 0.28 | 0.42 | 0.53 | 0.27 | 0.88 | 0.37 | **1.88** | 0.41 | 0.28 | 0.35 | 0.30 | 0.43 | 0.49 | 0.45 | **0.97** | 0.25 | 0.58 | **F** |  |  |
| **F** | 3.19 | **4.59** | 1.21 | 1.31 | 2.53 | 0.37 | 0.27 | 0.55 | 0.42 | **1.10** | 0.25 | 0.38 | 0.43 | 0.31 | 0.44 | 0.56 | 0.52 | 0.70 | 0.21 | 0.47 | **G** |  |  |
| **G** | 2.27 | 2.54 | 1.20 | 0.94 | 2.27 | 1.12 | 0.09 | 0.31 | 0.14 | 0.58 | 0.14 | 0.14 | 0.21 | 0.16 | 0.21 | 0.24 | 0.22 | 0.32 | 0.09 | 0.20 | **H** |  |  |
| **H** | 2.04 | 3.44 | 1.77 | 1.31 | 2.73 | 1.92 | 1.48 | 0.86 | 0.50 | **3.23** | 0.55 | 0.33 | 0.39 | 0.41 | 0.51 | 0.59 | 0.69 | **1.58** | 0.26 | 0.70 | **I** |  |  |
| **I** | 3.26 | **4.42** | 0.99 | 1.32 | **3.98** | 1.76 | 2.38 | 2.91 | 0.11 | **0.96** | 0.19 | 0.24 | 0.23 | 0.22 | 0.20 | 0.38 | 0.32 | 0.47 | 0.13 | 0.36 | **K** |  |  |
| **K** | 1.13 | 1.51 | 1.21 | 1.12 | 1.41 | 1.13 | 0.93 | 1.42 | 0.28 | **3.25** | **1.01** | 0.58 | 0.79 | 0.74 | **1.07** | **1.12** | **1.27** | **3.25** | 0.52 | **1.35** | **L** |  |  |
| **L** | 3.50 | **4.28** | 0.98 | 1.31 | **4.24** | 1.76 | 2.22 | **5.46** | 1.38 | 2.75 | 0.11 | 0.14 | 0.17 | 0.17 | 0.22 | 0.27 | 0.25 | 0.55 | 0.11 | 0.31 | **M** |  |  |
| **M** | 2.91 | 3.64 | 0.99 | 1.19 | **4.04** | 1.79 | 2.27 | **4.05** | 1.18 | **3.74** | 1.87 | 0.15 | 0.25 | 0.21 | 0.25 | 0.36 | 0.32 | 0.36 | 0.10 | 0.24 | **N** |  |  |
| **N** | 1.55 | 1.80 | 1.21 | 0.92 | 1.69 | 1.64 | 1.47 | 1.49 | 0.94 | 1.32 | 1.34 | 0.90 | 0.15 | 0.25 | 0.30 | 0.34 | 0.36 | 0.49 | 0.15 | 0.34 | **P** |  |  |
| **P** | 1.79 | 2.54 | 1.40 | 1.12 | 2.20 | 1.94 | 2.30 | 1.84 | 0.94 | 1.87 | 1.73 | 1.57 | 0.98 | 0.12 | 0.22 | 0.31 | 0.31 | 0.41 | 0.11 | 0.24 | **Q** |  |  |
| **Q** | 1.45 | 1.92 | 0.87 | 0.85 | 1.67 | 1.23 | 1.46 | 1.69 | 0.77 | 1.55 | 1.57 | 1.18 | 1.48 | 0.59 | 0.13 | 0.42 | 0.35 | 0.56 | 0.16 | 0.36 | **R** |  |  |
| **R** | 1.52 | 2.08 | 1.64 | 1.43 | 1.94 | 1.43 | 1.58 | 1.73 | 0.57 | 1.82 | 1.65 | 1.12 | 1.45 | 0.92 | 0.46 | 0.29 | 0.46 | 0.64 | 0.19 | 0.39 | **S** |  |  |
| **S** | 1.83 | 2.35 | 1.21 | 1.03 | 2.06 | 1.68 | 1.70 | 1.87 | 1.02 | 1.78 | 1.87 | 1.51 | 1.53 | 1.22 | 1.34 | 0.85 | 0.24 | 0.73 | 0.18 | 0.37 | **T** |  |  |
| **T** | 2.18 | 2.53 | 1.24 | 1.08 | 2.28 | 1.86 | 1.89 | 2.59 | 1.02 | 2.40 | 2.05 | 1.62 | 1.90 | 1.45 | 1.34 | 1.63 | 0.99 | 0.82 | 0.28 | 0.69 | **V** |  |  |
| **V** | 3.35 | **3.98** | 1.13 | 1.20 | **4.00** | 2.03 | 2.21 | **4.85** | 1.23 | **5.00** | 3.70 | 1.50 | 2.11 | 1.56 | 1.74 | 1.84 | 2.51 | 2.31 | 0.04 | 0.18 | **W** |  |  |
| **W** | 2.86 | 3.24 | 1.51 | 1.28 | **4.26** | 2.56 | 2.50 | 3.39 | 1.40 | 3.37 | 3.02 | 1.75 | 2.75 | 1.67 | 2.05 | 2.33 | 2.59 | 3.33 | 1.97 | 0.22 | **Y** |  |  |
| **Y** | 3.08 | **4.06** | 1.36 | 1.33 | **4.18** | 2.43 | 2.51 | **3.82** | 1.67 | 3.69 | **3.71** | 1.75 | 2.63 | 1.63 | 1.99 | 2.00 | 2.23 | 3.41 | **3.83** | 1.95 |  |  |  |
| **f_pn_** | **A** | **C** | **D** | **E** | **F** | **G** | **H** | **I** | **K** | **L** | **M** | **N** | **P** | **Q** | **R** | **S** | **T** | **V** | **W** | **Y** |  | |  |

Table B.2 Parameters *f_p_* (Upper Half) and *f_pn_* (Lower Half) for all amino acid pairs in proteins from class Beta. Top 20 residue pairs with the highest values are in bold and the 20 residue pairs with the lowest values are underlined. Here the cutoff values is 8 Å and the separation value is 10

|  | **A** | **C** | **D** | **E** | **F** | **G** | **H** | **I** | **K** | **L** | **M** | **N** | **P** | **Q** | **R** | **S** | **T** | **V** | **W** | **Y** | **f_p_** |
| --- | --- | --- | --- | --- | --- | --- | --- | --- | --- | --- | --- | --- | --- | --- | --- | --- | --- | --- | --- | --- | --- |
|  | 0.56 | 0.28 | 0.53 | 0.47 | 0.81 | **1.14** | 0.26 | **1.05** | 0.50 | **1.41** | 0.28 | 0.51 | 0.57 | 0.38 | 0.52 | 0.81 | 0.84 | **1.49** | 0.29 | 0.64 | **A** |
| **A** | 1.36 | 0.33 | 0.15 | 0.17 | 0.26 | 0.33 | 0.11 | 0.31 | 0.19 | 0.45 | 0.09 | 0.16 | 0.19 | 0.15 | 0.18 | 0.30 | 0.27 | 0.47 | 0.12 | 0.21 | **C** |
| **C** | 2.53 | **11.17** | 0.15 | 0.23 | 0.32 | 0.68 | 0.21 | 0.42 | 0.55 | 0.51 | 0.12 | 0.44 | 0.35 | 0.24 | 0.48 | 0.54 | 0.51 | 0.60 | 0.13 | 0.31 | **D** |
| **D** | 1.40 | 1.50 | 0.44 | 0.12 | 0.34 | 0.57 | 0.19 | 0.49 | 0.61 | 0.63 | 0.14 | 0.35 | 0.35 | 0.24 | 0.52 | 0.49 | 0.53 | 0.75 | 0.13 | 0.33 | **E** |
| **E** | 1.26 | 1.70 | 0.67 | 0.35 | 0.38 | 0.68 | 0.22 | 0.94 | 0.38 | **1.19** | 0.25 | 0.34 | 0.41 | 0.30 | 0.40 | 0.60 | 0.59 | **1.22** | 0.25 | 0.56 | **F** |
| **F** | 3.07 | **3.61** | 1.32 | 1.42 | 2.24 | 0.67 | 0.34 | 0.83 | 0.61 | **1.11** | 0.25 | 0.73 | 0.68 | 0.47 | 0.60 | **0.99** | 0.95 | **1.25** | 0.26 | 0.64 | **G** |
| **G** | 2.19 | 2.37 | 1.44 | 1.21 | 2.05 | 1.01 | 0.07 | 0.27 | 0.16 | 0.37 | 0.09 | 0.16 | 0.18 | 0.13 | 0.18 | 0.29 | 0.27 | 0.36 | 0.09 | 0.20 | **H** |
| **H** | 1.83 | 2.97 | 1.64 | 1.50 | 2.47 | 1.92 | 1.53 | 0.89 | 0.56 | **1.88** | 0.34 | 0.42 | 0.46 | 0.39 | 0.51 | 0.73 | 0.86 | **2.09** | 0.29 | 0.76 | **I** |
| **I** | 2.95 | 3.18 | 1.31 | 1.53 | **4.12** | 1.85 | 2.20 | 2.87 | 0.15 | 0.65 | 0.14 | 0.37 | 0.32 | 0.27 | 0.25 | 0.54 | 0.56 | 0.79 | 0.16 | 0.40 | **K** |
| **K** | 1.35 | 1.86 | 1.62 | 1.82 | 1.60 | 1.31 | 1.24 | 1.75 | 0.45 | **1.24** | 0.41 | 0.52 | 0.66 | 0.51 | 0.70 | **0.96** | **1.07** | **2.59** | 0.44 | **0.95** | **L** |
| **L** | 2.96 | **3.49** | 1.16 | 1.45 | **3.91** | 1.84 | 2.24 | **4.58** | 1.52 | 2.25 | 0.06 | 0.14 | 0.15 | 0.11 | 0.14 | 0.21 | 0.22 | 0.45 | 0.09 | 0.20 | **M** |
| **M** | 2.57 | 2.94 | 1.19 | 1.42 | **3.55** | 1.81 | 2.30 | **3.53** | 1.42 | 3.23 | 1.96 | 0.26 | 0.39 | 0.28 | 0.30 | 0.58 | 0.57 | 0.59 | 0.14 | 0.32 | **N** |
| **N** | 1.63 | 1.84 | 1.53 | 1.21 | 1.66 | 1.83 | 1.49 | 1.54 | 1.28 | 1.42 | 1.60 | 1.09 | 0.23 | 0.25 | 0.31 | 0.53 | 0.52 | 0.68 | 0.21 | 0.41 | **P** |
| **P** | 1.76 | 2.13 | 1.19 | 1.18 | 1.95 | 1.64 | 1.63 | 1.62 | 1.09 | 1.77 | 1.75 | 1.58 | 0.91 | 0.10 | 0.25 | 0.41 | 0.42 | 0.55 | 0.12 | 0.28 | **Q** |
| **Q** | 1.60 | 2.37 | 1.13 | 1.10 | 1.99 | 1.58 | 1.66 | 1.89 | 1.28 | 1.88 | 1.77 | 1.52 | 1.35 | 0.76 | 0.13 | 0.51 | 0.49 | 0.74 | 0.20 | 0.39 | **R** |
| **R** | 1.79 | 2.30 | 1.79 | 1.98 | 2.15 | 1.63 | 1.75 | 2.01 | 0.96 | 2.07 | 1.82 | 1.33 | 1.37 | 1.50 | 0.63 | 0.45 | 0.88 | **1.06** | 0.25 | 0.50 | **S** |
| **S** | 1.76 | 2.38 | 1.28 | 1.16 | 2.02 | 1.70 | 1.84 | 1.84 | 1.29 | 1.81 | 1.71 | 1.65 | 1.46 | 1.55 | 1.56 | 0.88 | 0.50 | **1.26** | 0.22 | 0.52 | **T** |
| **T** | 1.97 | 2.34 | 1.31 | 1.35 | 2.13 | 1.74 | 1.84 | 2.33 | 1.44 | 2.15 | 1.92 | 1.73 | 1.53 | 1.72 | 1.61 | 1.83 | 1.11 | **1.47** | 0.38 | **0.99** | **V** |
| **V** | 2.99 | **3.48** | 1.32 | 1.65 | **3.83** | 1.98 | 2.08 | **4.84** | 1.74 | **4.48** | 3.37 | 1.54 | 1.73 | 1.91 | 2.11 | 1.90 | 2.42 | 2.42 | 0.05 | 0.22 | **W** |
| **W** | 2.71 | **4.13** | 1.29 | 1.36 | **3.60** | 1.94 | 2.33 | 3.10 | 1.62 | **3.59** | 3.07 | 1.74 | 2.44 | 2.00 | 2.58 | 2.10 | 1.96 | 2.95 | 1.82 | 0.26 | **Y** |
| **Y** | 2.66 | 3.23 | 1.43 | 1.49 | **3.61** | 2.10 | 2.41 | **3.64** | 1.83 | **3.43** | 3.11 | 1.72 | 2.16 | 2.06 | 2.31 | 1.85 | 2.08 | **3.38** | **3.47** | 1.85 |  |
| **f_pn_** | **A** | **C** | **D** | **E** | **F** | **G** | **H** | **I** | **K** | **L** | **M** | **N** | **P** | **Q** | **R** | **S** | **T** | **V** | **W** | **Y** |  |

Table B.3 Parameters *f_p_* (Upper Half) and *f_pn_* (Lower Half) for all amino acid pairs in proteins from class Alpha+Beta. Top 20 residue pairs with the highest values are in bold and the 20 residue pairs with the lowest values are underlined. Here the cutoff values is 8 Å and the separation value is 10

|  | **A** | **C** | **D** | **E** | **F** | **G** | **H** | **I** | **K** | **L** | **M** | **N** | **P** | **Q** | **R** | **S** | **T** | **V** | **W** | **Y** | **f_p_** |
| --- | --- | --- | --- | --- | --- | --- | --- | --- | --- | --- | --- | --- | --- | --- | --- | --- | --- | --- | --- | --- | --- |
|  | **1.00** | 0.30 | 0.63 | 0.63 | **0.96** | **1.42** | 0.39 | **1.68** | 0.56 | **2.36** | 0.49 | 0.51 | 0.68 | 0.40 | 0.63 | 0.85 | 0.94 | **2.20** | 0.25 | 0.67 | **A** |
| **A** | 1.38 | 0.14 | 0.12 | 0.12 | 0.21 | 0.27 | 0.11 | 0.29 | 0.12 | 0.43 | 0.10 | 0.12 | 0.14 | 0.08 | 0.12 | 0.18 | 0.19 | 0.37 | 0.06 | 0.15 | **C** |
| **C** | 2.72 | **8.16** | 0.17 | 0.26 | 0.30 | 0.69 | 0.27 | 0.47 | 0.54 | 0.61 | 0.18 | 0.35 | 0.37 | 0.21 | 0.50 | 0.51 | 0.48 | 0.61 | 0.11 | 0.28 | **D** |
| **D** | 1.28 | 1.60 | 0.49 | 0.13 | 0.34 | 0.57 | 0.22 | 0.52 | 0.52 | 0.71 | 0.17 | 0.29 | 0.35 | 0.19 | 0.51 | 0.45 | 0.45 | 0.66 | 0.10 | 0.28 | **E** |
| **E** | 1.07 | 1.28 | 0.64 | 0.26 | 0.32 | 0.66 | 0.23 | **0.94** | 0.34 | **1.31** | 0.30 | 0.28 | 0.38 | 0.23 | 0.36 | 0.46 | 0.47 | **1.14** | 0.18 | 0.45 | **F** |
| **F** | 2.84 | **3.91** | 1.30 | 1.21 | 1.99 | 0.70 | 0.37 | 0.94 | 0.55 | **1.31** | 0.35 | 0.58 | 0.66 | 0.37 | 0.62 | 0.85 | 0.85 | **1.28** | 0.20 | 0.54 | **G** |
| **G** | 2.19 | 2.69 | 1.53 | 1.07 | 2.15 | 1.19 | 0.08 | 0.31 | 0.14 | 0.45 | 0.12 | 0.17 | 0.20 | 0.12 | 0.18 | 0.29 | 0.28 | 0.40 | 0.08 | 0.18 | **H** |
| **H** | 1.91 | **3.39** | 1.93 | 1.37 | 2.40 | 2.03 | 1.37 | **1.10** | 0.51 | **2.61** | 0.49 | 0.40 | 0.53 | 0.33 | 0.51 | 0.66 | 0.86 | **2.42** | 0.22 | 0.70 | **I** |
| **I** | 3.31 | **3.68** | 1.34 | 1.25 | **3.94** | 2.05 | 2.17 | 3.09 | 0.13 | 0.68 | 0.15 | 0.27 | 0.27 | 0.18 | 0.20 | 0.37 | 0.39 | 0.65 | 0.10 | 0.31 | **K** |
| **K** | 1.13 | 1.51 | 1.59 | 1.29 | 1.45 | 1.24 | 1.05 | 1.48 | 0.38 | **1.86** | 0.63 | 0.52 | 0.83 | 0.47 | 0.75 | 0.92 | **1.09** | **3.14** | 0.33 | **0.94** | **L** |
| **L** | 3.00 | **3.51** | 1.13 | 1.10 | **3.53** | 1.85 | 2.06 | **4.71** | 1.27 | 2.16 | 0.08 | 0.15 | 0.20 | 0.11 | 0.17 | 0.23 | 0.25 | 0.59 | 0.08 | 0.21 | **M** |
| **M** | 2.61 | 3.29 | 1.36 | 1.11 | **3.42** | 2.08 | 2.29 | **3.75** | 1.17 | 3.06 | 1.61 | 0.19 | 0.29 | 0.20 | 0.25 | 0.42 | 0.40 | 0.48 | 0.10 | 0.24 | **N** |
| **N** | 1.48 | 2.16 | 1.46 | 1.03 | 1.72 | 1.86 | 1.73 | 1.64 | 1.14 | 1.38 | 1.61 | 1.11 | 0.19 | 0.23 | 0.35 | 0.42 | 0.42 | 0.66 | 0.14 | 0.35 | **P** |
| **P** | 1.76 | 2.36 | 1.39 | 1.13 | 2.12 | 1.90 | 1.84 | 1.95 | 1.04 | 1.97 | 1.97 | 1.55 | 0.94 | 0.07 | 0.20 | 0.27 | 0.28 | 0.42 | 0.08 | 0.19 | **Q** |
| **Q** | 1.34 | 1.73 | 1.04 | 0.79 | 1.64 | 1.38 | 1.44 | 1.57 | 0.91 | 1.45 | 1.48 | 1.41 | 1.43 | 0.58 | 0.14 | 0.40 | 0.42 | 0.66 | 0.12 | 0.30 | **R** |
| **R** | 1.45 | 1.83 | 1.68 | 1.44 | 1.77 | 1.59 | 1.47 | 1.68 | 0.69 | 1.59 | 1.52 | 1.23 | 1.50 | 1.13 | 0.54 | 0.30 | 0.58 | 0.85 | 0.14 | 0.36 | **S** |
| **S** | 1.78 | 2.47 | 1.55 | 1.16 | 2.06 | 1.99 | 2.16 | 1.98 | 1.15 | 1.76 | 1.85 | 1.81 | 1.66 | 1.39 | 1.41 | 0.97 | 0.32 | **1.08** | 0.13 | 0.37 | **T** |
| **T** | 2.11 | 2.73 | 1.58 | 1.25 | 2.25 | 2.12 | 2.26 | 2.76 | 1.28 | 2.24 | 2.21 | 1.89 | 1.77 | 1.55 | 1.56 | 1.99 | 1.17 | **1.54** | 0.27 | 0.79 | **V** |
| **V** | **3.48** | **3.81** | 1.39 | 1.28 | **3.82** | 2.25 | 2.27 | **5.45** | 1.50 | **4.54** | **3.59** | 1.58 | 1.94 | 1.62 | 1.73 | 2.02 | 2.78 | 2.77 | 0.04 | 0.13 | **W** |
| **W** | 2.29 | **3.39** | 1.42 | 1.18 | **3.50** | 2.08 | 2.48 | 2.85 | 1.30 | 2.82 | 2.69 | 1.84 | 2.36 | 1.75 | 1.90 | 1.90 | 2.00 | 2.82 | 2.17 | 0.18 | **Y** |
| **Y** | 2.36 | 3.32 | 1.44 | 1.22 | **3.36** | 2.11 | 2.31 | **3.52** | 1.58 | 3.02 | 2.86 | 1.77 | 2.30 | 1.63 | 1.78 | 1.92 | 2.12 | 3.18 | 3.07 | 1.57 |  |
| **f_pn_** | **A** | **C** | **D** | **E** | **F** | **G** | **H** | **I** | **K** | **L** | **M** | **N** | **P** | **Q** | **R** | **S** | **T** | **V** | **W** | **Y** |  |
