# Supplementary material for: Characteristics of protein residue-residue contacts and their application in contact prediction
Source: J Mol Model. 2014 Nov 6;20(11):2497. doi: 10.1007/s00894-014-2497-9 (PMC4221654; doi:10.1007/s00894-014-2497-9)
Supplement: Supplementary file 3 — (DOCX 20 kb) [file 894_2014_2497_MOESM3_ESM.docx]

**Appendix C**

**Table C.1** Values of observed frequency for the top 20 interacting pairs for membrane and soluble proteins from Adamian & Liang (2001) with *f_p_* values of these pairs calculated in our study for proteins from classes Alpha and Beta. Here the *cutoff* value is 8 Å and the *separation* value is 10. Pairs which occur in our top 20 pairs with the highest *f_p_* values are denoted in bold

| **Amino acid pair** | **f_p_ [%]** | | |  | **Amino acid pair** | **f_p_ [%]** | | |
| --- | --- | --- | --- | --- | --- | --- | --- | --- |
|  | **Membrane**  **proteins** | **Alpha** | **Beta** |  |  | **Soluble**  **proteins** | **Alpha** | **Beta** |
| L-F | 4.37 | **1.88** | **1.19** |  | L-L | 3.41 | **3.25** | **1.24** |
| L-L | 2.93 | **3.25** | **1.24** |  | I-L | 3.09 | **3.23** | **1.88** |
| L-V | 2.78 | **3.25** | **2.59** |  | L-F | 2.99 | **1.88** | **1.19** |
| I-L | 2.72 | **3.23** | **1.88** |  | L-V | 2.91 | **3.25** | **2.59** |
| F-W | 2.61 | 0.25 | 0.25 |  | A-L | 2.88 | **3.33** | **1.41** |
| L-W | 2.52 | 0.52 | 0.44 |  | L-Y | 2.65 | **1.35** | **0.95** |
| F-F | 2.50 | 0.42 | 0.38 |  | L-R | 1.94 | **1.07** | 0.70 |
| A-L | 2.03 | **3.33** | **1.41** |  | L-M | 1.66 | **1.01** | 0.41 |
| L-Y | 1.98 | **1.35** | **0.95** |  | G-L | 1.38 | **1.10** | **1.11** |
| F-V | 1.88 | **0.97** | **1.22** |  | A-V | 1.33 | **1.75** | **1.49** |
| F-I | 1.75 | 0.88 | 0.94 |  | F-I | 1.30 | 0.88 | 0.94 |
| A-F | 1.73 | **1.14** | 0.81 |  | L-W | 1.24 | 0.52 | 0.44 |
| M-F | 1.61 | 0.41 | 0.25 |  | F-Y | 1.24 | 0.58 | 0.56 |
| L-M | 1.57 | **1.01** | 0.41 |  | L-T | 1.22 | **1.27** | **1.07** |
| L-S | 1.38 | **1.12** | **0.96** |  | A-I | 1.22 | **1.56** | **1.05** |
| I-W | 1.33 | 0.26 | 0.29 |  | K-L | 1.17 | **0.96** | 0.65 |
| I-V | 1.21 | **1.58** | **2.09** |  | A-F | 1.13 | **1.14** | 0.81 |
| G-L | 1.20 | **1.10** | **1.11** |  | E-R | 1.13 | 0.57 | 0.52 |
| W-V | 1.17 | 0.28 | 0.38 |  | F-V | 1.12 | **0.97** | **1.22** |
| G-F | 1.15 | 0.53 | 0.68 |  | R-Y | 1.11 | 0.36 | 0.39 |
